# Phosphoproteomic Profiling Reveals Epstein-Barr Virus Protein Kinase Integration of DNA Damage Response and Mitotic Signaling

**DOI:** 10.1371/journal.ppat.1005346

**Published:** 2015-12-29

**Authors:** Renfeng Li, Gangling Liao, Raja Sekhar Nirujogi, Sneha M. Pinto, Patrick G. Shaw, Tai-Chung Huang, Jun Wan, Jiang Qian, Harsha Gowda, Xinyan Wu, Dong-Wen Lv, Kun Zhang, Srikanth S. Manda, Akhilesh Pandey, S. Diane Hayward

**Affiliations:** 1 Department of Oncology, Johns Hopkins University School of Medicine, Baltimore, Maryland, United States of America; 2 Philips Institute for Oral Health Research, VCU School of Dentistry, Virginia Commonwealth University, Richmond, Virginia, United States of America; 3 Massey Cancer Center, Virginia Commonwealth University, Richmond, Virginia, United States of America; 4 McKusick-Nathans Institute of Genetic Medicine, Johns Hopkins University School of Medicine, Baltimore, Maryland, United States of America; 5 Institute of Bioinformatics, International Technology Park, Bangalore, India; 6 Wilmer Institute, Johns Hopkins University School of Medicine, Baltimore, Maryland, United States of America; 7 Diana Helis Henry Medical Research Foundation, New Orleans, Louisiana, United States of America; Baylor College of Medicine, UNITED STATES

## Abstract

Epstein-Barr virus (EBV) is etiologically linked to infectious mononucleosis and several human cancers. EBV encodes a conserved protein kinase BGLF4 that plays a key role in the viral life cycle. To provide new insight into the host proteins regulated by BGLF4, we utilized stable isotope labeling by amino acids in cell culture (SILAC)-based quantitative proteomics to compare site-specific phosphorylation in BGLF4-expressing Akata B cells. Our analysis revealed BGLF4-mediated hyperphosphorylation of 3,046 unique sites corresponding to 1,328 proteins. Frequency analysis of these phosphosites revealed a proline-rich motif signature downstream of BGLF4, indicating a broader substrate recognition for BGLF4 than its cellular ortholog cyclin-dependent kinase 1 (CDK1). Further, motif analysis of the hyperphosphorylated sites revealed enrichment in ATM, ATR and Aurora kinase substrates while functional analyses revealed significant enrichment of pathways related to the DNA damage response (DDR), mitosis and cell cycle. Phosphorylation of proteins associated with the mitotic spindle assembly checkpoint (SAC) indicated checkpoint activation, an event that inactivates the anaphase promoting complex/cyclosome, APC/C. Furthermore, we demonstrated that BGLF4 binds to and directly phosphorylates the key cellular proteins PP1, MPS1 and CDC20 that lie upstream of SAC activation and APC/C inhibition. Consistent with APC/C inactivation, we found that BGLF4 stabilizes the expression of many known APC/C substrates. We also noted hyperphosphorylation of 22 proteins associated the nuclear pore complex, which may contribute to nuclear pore disassembly and SAC activation. A drug that inhibits mitotic checkpoint activation also suppressed the accumulation of extracellular EBV virus. Taken together, our data reveal that, in addition to the DDR, manipulation of mitotic kinase signaling and SAC activation are mechanisms associated with lytic EBV replication. All MS data have been deposited in the ProteomeXchange with identifier PXD002411 (http://proteomecentral.proteomexchange.org/dataset/PXD002411).

## Author Summary

Epstein-Barr virus (EBV) is a herpesvirus that is associated with B cell and epithelial human cancers. Herpesviruses encode a protein kinase which is an important regulator of lytic virus replication and is consequently a target for anti-viral drug development. The EBV genome encodes for a serine/threonine protein kinase called BGLF4. Previous work on BGLF4 has largely focused on its cyclin-dependent kinase 1 (CDK1)-like activity. The range of BGLF4 cellular substrates and the full impact of BGLF4 on the intracellular microenvironment still remain to be elucidated. Here, we utilized unbiased quantitative phosphoproteomic approach to dissect the changes in the cellular phosphoproteome that are mediated by BGLF4. Our MS analyses revealed extensive hyperphosphorylation of substrates that are normally targeted by CDK1, Ataxia telangiectasia mutated (ATM), Ataxia telangiectasia and Rad3-related (ATR) proteins and Aurora kinases. The up-regulated phosphoproteins were functionally linked to the DNA damage response, mitosis and cell cycle pathways. Our data demonstrate widespread changes in the cellular phosphoproteome that occur upon BGLF4 expression and suggest that manipulation of the DNA damage and mitotic kinase signaling pathways are central to efficient EBV lytic replication.

## Introduction

Infection with Epstein-Barr virus (EBV), a ubiquitous herpesvirus, is associated with malignant disease, including Burkitt lymphoma, nasopharyngeal carcinoma, gastric carcinoma, and post-transplant lymphoproliferative disease [1,2]. While EBV latency proteins drive proliferation, lytic EBV gene products have also been implicated in tumorigenesis [3,4]. The EBV protein kinase BGLF4, an early lytic gene product, is conserved across the order herpesviridae [5,6]. Due to its unique nature and key role in infectious virus production [7,8], BGLF4 and its downstream effectors are potentially druggable targets [6,9–11].

BGLF4 phosphorylates both viral and cellular proteins [6,12] to generate an environment suitable for efficient viral replication. BGLF4 phosphorylates EBV latency and lytic proteins to regulate their transactivation activity [13–15] and expression [16–18]. It also phosphorylates EBV encoded replication proteins to facilitate lytic DNA replication [19–21]. In addition, BGLF4 interacts with and phosphorylates host cellular proteins involved in DNA replication to block cellular chromosomal DNA replication [22], create a pseudo-S phase environment [23,24] and initiates a DNA damage response (DDR) beneficial for viral replication [6]. BGLF4 also phosphorylates host cellular proteins to alter microtubule dynamics [25], disrupt the nuclear lamina [24,26] and affect nuclear pore permeability [27]. Protein SUMOylation is modified by BGLF4 in a kinase activity and SUMO-binding dependent manner [28,29] and BGLF4 phosphorylates IRF3 and UXT to suppress host immune responses and NF-κB signaling, respectively [30–32]. Taken together, these studies indicate that BGLF4 affects many aspects of the cellular environment. However, a global overview of BGLF4 regulated signaling events in cells is still lacking.

Recent advances in proteomics have enabled the discovery of potential kinase substrates *in vitro* and *in vivo* [6,33–35]. In particular, quantitative phosphoproteomics based on stable isotope labeling by amino acids in cell culture (SILAC) has greatly facilitated the elucidation of global phosphorylation regulation in signaling pathways mediated by TSLP [36], IL-33 [37], oncogenic PI3KCA mutations [34], DDR and mitosis pathways [38–40]. In this study, we employed a SILAC-based quantitative phosphoproteomic approach to dissect BGLF4-regulated downstream signaling events. We quantified 7,568 unique phosphorylation sites including 3,046 hyperphosphorylated sites in 1,328 proteins. Bioinformatics and network analyses revealed that BGLF4 regulates many proteins involved in the modulation of the DDR, mitosis and nuclear transport, in part through regulation of cellular kinase and phosphatase activity. Integration of protein phosphorylation events predicted activation of the spindle assembly checkpoint (SAC) by BGLF4 and hence the inhibition of the anaphase promoting complex/cyclosome (APC/C) E3 ubiquitin ligase activity. Parallel proteomic analysis of the nuclear proteome revealed increased protein levels for multiple known APC/C substrates and this was confirmed by western blot analysis. This study significantly expands the information on host protein phosphorylation regulated by the EBV conserved protein kinase and provides a foundation for the development of new anti-herpesvirus strategies.

## Results

### Mass spectrometry (MS) analysis of BGLF4-induced changes in the cell phosphoproteome and proteome

Akata (EBV+) B cell lines modified to express doxycycline inducible BGLF4 or vector control [21] were used to examine the changes in protein expression and phosphorylation levels that occurred subsequent to doxycycline induction of BGLF4 expression. Akata (EBV+)-BGLF4 cells were grown in light medium while vector control Akata (EBV+) cells were grown in medium supplemented with heavy isotopes of lysine and arginine (^13^C_6_, ^15^N_2_-lysine and ^13^C_6_, ^15^N_4_-arginine). Doxycycline was added for 48 hours prior to harvesting. Western blotting with antibody that recognizes phospho-serine in the context of a CDK substrate motif ([K/R]-S-P-X-[K/R]) confirmed that BGLF4 induced changes in protein phosphorylation under these conditions (S1 Fig, upper panel). An increase in γ-H2AX protein, a known downstream result of BGLF4 expression, was also documented at 48 hr along with induction of BGLF4 itself (S1 Fig, lower panel). In contrast, there was no obvious change in the overall pattern of phosphorylation of protein kinase A (PKA) substrates as detected by western blotting with an antibody that detects phospho-serine or -threonine in the context of RRxS/T (S1 Fig, middle panel).

**Fig 1:**
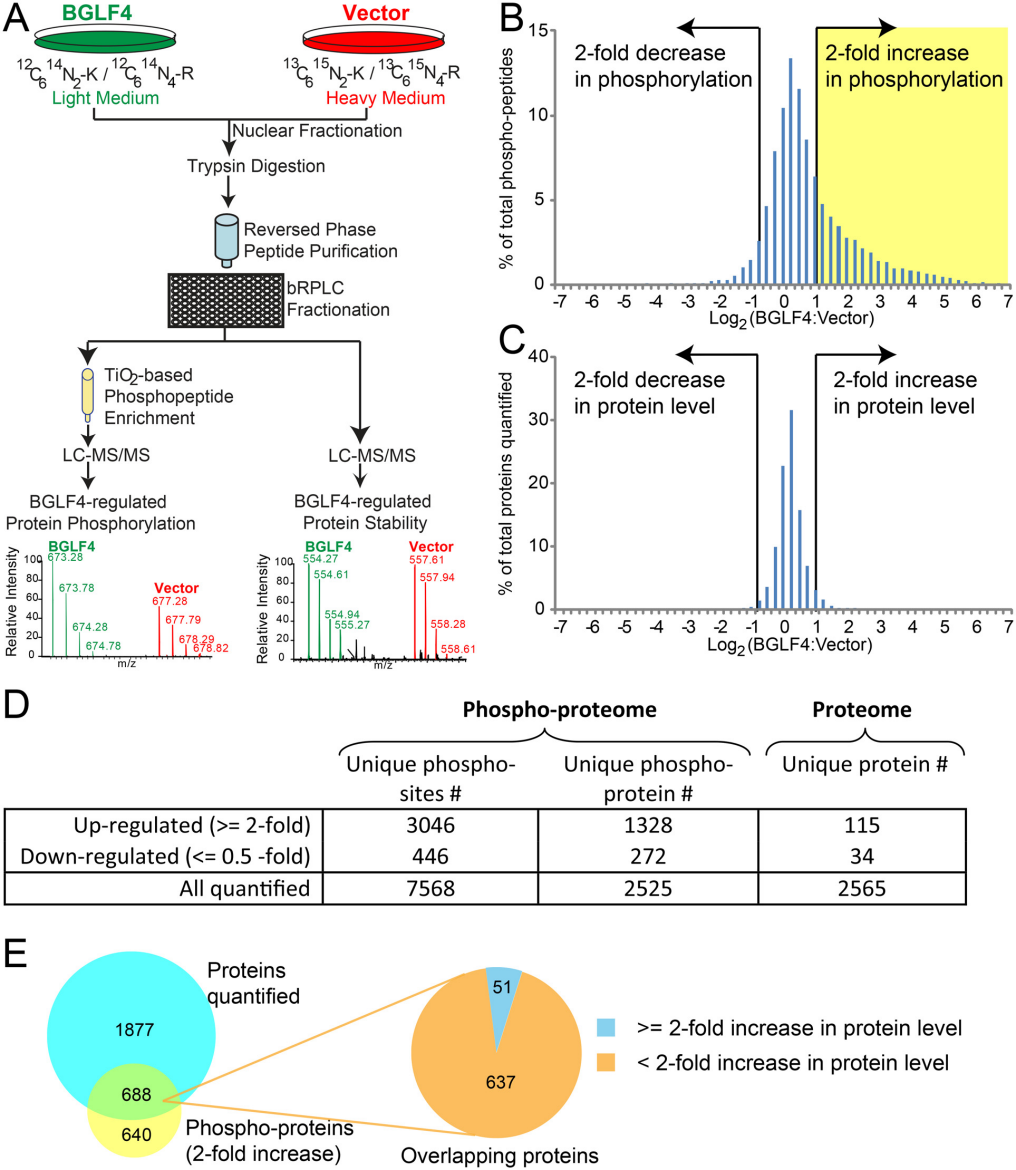
Proteomic analysis of BGLF4-induced protein phosphorylation. (A) Schematic illustration of the SILAC-based quantitative proteomic approach. Akata (EBV+)-tet-BGLF4 and Akata (EBV+)-tet-Vector cells were cultured in “light” (^12^C_6_^14^N_2_-K and ^12^C_6_^14^N_4_-R) and “heavy” (^13^C_6_^15^N_2_-K and ^13^C_6_^15^N_4_-R) medium respectively. The cells were then treated with doxycycline for 48 hrs and equal amounts of cells were mixed, fractionated and the nuclear fractions were lysed, digested, desalted and then subjected to lyophilization. The peptide mixture was further fractionated by bRPLC and concatenated to 12 fractions. One peptide aliquot was used for phosphopeptide enrichment by TiO_2_ beads and the other was used for protein level analysis. The samples were analyzed by LC-MS/MS and the resulting high resolution mass spectra revealed BGLF4-induced changes in phosphorylation level and protein level. See also S1 Fig. (B and C) Quantitation of phosphopeptide and protein levels after BGLF4 induction. Log_2_ (BGLF4:Vector) plots for quantified phosphopeptides (B) and protein level (C) for Akata (EBV+)-tet-BGLF4 vs Akata (EBV+)-tet-Vector cells. See also S1 and S2 Tables. (D) Summary of phosphoproteomic and proteomic data obtained in the MS analysis (E) Overlap of up-regulated phosphoproteins (> = 2-fold increase) and total proteins quantified.

For MS analysis, nitrogen cavitation was carried out [41] and the nuclear material was lysed and digested with trypsin. The tryptic peptides were further fractionated using high pH reversed-phase liquid chromatography (bRPLC) and 12 fractions were collected. Phosphopeptides were enriched and analyzed on an LTQ-Orbitrap Elite mass spectrometer. For cell nuclear proteome analysis, 12 fractions were analyzed on an LTQ-Orbitrap Velos mass spectrometer. Protein and phosphorylation site identification and quantitation were carried out using Thermo Proteome Discoverer (PD 2.0) software suite (Fig 1A). Statistical analysis of quantified phosphopeptides showed a good correlation between two biological replicates (S2 Fig). A total of 7,568 unique phosphosites from 2,525 proteins were quantified in the MS analyses (S1 Table). For the nuclear proteome, a total of 2,565 proteins were quantified (S2 Table).

Phosphopeptides that showed a 2-fold or greater change in BGLF4 expressing Akata (EBV+) cells versus vector control Akata (EBV+) cells were considered to be hyper or hypophosphorylated, while those that changed less than 1.2-fold were considered as unregulated. Comparison of the light: heavy ratios of the detected phosphopeptides revealed that 3,046 unique phosphosites (40%) were hyperphosphorylated in BGLF4-expressing cells (Fig 1B, 1D and S1 Table). In contrast, only 115 out of 2,565 quantified proteins (4%) were overexpressed by 2-fold or more in the same condition (Fig 1C, 1D and S2 Table). The hyperphosphorylated phosphopeptides correlate to 1,328 unique proteins. For 688 of the 1,328 proteins, information was also obtained on protein levels. Of these 688, 93% showed less than 2-fold increase in total protein level (Fig 1E). Thus increased phosphorylation, in general, represented an increase in post-translational modification in the absence of a change in protein level.

### Impact of BGLF4 on cell kinase and phosphatase activity

**Fig 2:**
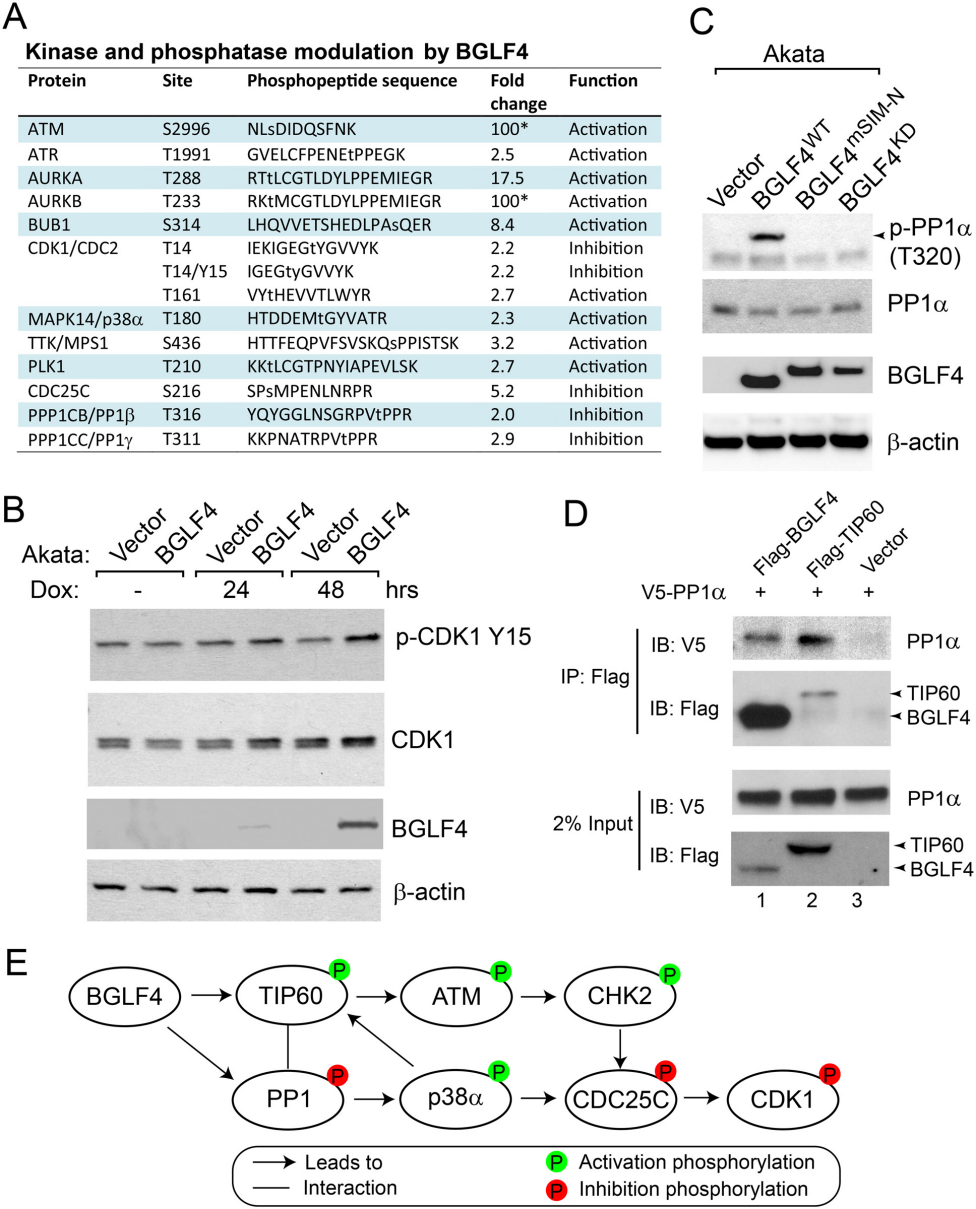
BGLF4-induced kinase and phosphatase phosphorylation. (A) Examples of kinase and phosphatase modulation by BGLF4. *: The maximum BGLF4/Vector ratio was set as 100-fold. (B) Western blot analysis of cell extracts from Dox inducible Akata (EBV+) cells carrying empty vector or BGLF4 using anti-phospho-CDK1, anti-CDK1, anti-HA (BGLF4) and anti-β-actin antibodies as indicated. The cells were untreated (-) or treated with doxycycline (Dox) for 24 hrs or 48 hrs. (C) Immunoblot analysis of cell lysates from Dox inducible Akata cells carrying empty vector, wild type BGLF4 (BGLF4^WT^), SUMO binding-deficient mutant (BGLF4^mSIM-N^), or kinase-dead mutant (BGLF4^KD^) using the antibodies indicated. Cells were treated with doxycycline for 48 hrs to induce BGLF4 expression. (D) Immunoprecipitation assay performed on extracts from transfected cells showing co-precipitation of V5-PP1α with Flag-BGLF4 (lane 1) and with Flag-TIP60 (lane 2). In the control cells V5-PP1α was co-transfected with Flag-vector (lane 3). (E) Summary of a potential BGLF4-triggered signaling cascade.

The activities of cell kinases and their counterbalancing phosphatases are regulated by post-translational modifications. BGLF4 therefore has the potential to indirectly manipulate the cellular environment by activation or repression of specific members of these protein families. A known example is BGLF4-induced phosphorylation and activation of the acetyl transferase TIP60 that then acetylates and activates the ATM kinase [6]. In the current MS analysis, increased phosphorylation at ATM S2996 was detected (Figs 2A and S3). This is a site of ATM auto-phosphorylation and hence a mark of ATM activity [42]. Phosphorylation of T180, detected in our screen (Fig 2A), is one of two activating phosphorylation sites on MAPK14/p38α that is introduced by the upstream MEK kinase following various cellular stresses including DNA damage and viral protein expression [43–45]. The activation of CHK2 and p38α can lead to the phosphorylation of phosphatase CDC25C at S216 [45,46], which was also detected in our current analysis (Fig 2A). The function of the other key DNA damage signaling regulator, ATR, is also modified during herpesvirus infections. ATR down-stream signaling is blocked in latently EBV infected cells by STAT3 [47] and in herpes simplex -1 infected cells undergoing lytic replication, ATR signaling is inhibited by the viral helicase-primase complex although ATR pathway proteins are involved in the replication process itself [48,49]. We detected increased phosphorylation of ATR (Figs 2A and S3), consistent with activated phosphorylation [50].

Y15 and T14 are inhibitory phosphorylation sites of CDK1 while singly phosphorylated T161 is an active form of CDK1 [51]. The most abundant form of CDK1 detected at S/G2 and G2/M is triple phosphorylated and is inactive. The phosphorylation status of CDK1 in cells is balanced by kinases (WEE1 and MYT1) and phosphatase CDC25C. The phosphorylation and inhibition of CDC25C might contribute to the increased phosphorylation of CDK1. Consistent with CDC25 inhibition, increased phosphorylation of CDK1 was detected at Y15, T14 and T161 after BGLF4 expression (Figs 2A and S3). This suggests that the majority of the CDK1 in BGLF4 expressing cells is in an inactive form. In addition to CDC25C, we also detected increased phosphorylation of protein phosphatase 1 (PP1) (Fig 2A). PP1 is normally inactivated by CDK1 mediated phosphorylation [52,53]. The inactivation site in PP1β is T316, in PP1γ is T311 and in PP1α is T320. Inactivation of PP1 would strengthen kinase signaling by removing a component of negative feedback control. Interestingly, in one of the PP1 targeting partners, CDCA2/RepoMan, we observed hyperphosphorylation at two sites (T423 and S429) that are in or adjacent to the RVxF PP1 binding site on CDCA2, locations that are known to inhibit PP1 binding [54] and would thus also perturb PP1 substrate targeting.

Western blot analyses were performed to confirm the presence of inactivating phosphorylation sites in CDK1 and PP1. In the case of PP1, PP1α was used to illustrate that all PP1 isoforms were subject to regulation by BGLF4. Akata (EBV+)-BGLF4 and Akata (EBV+)-vector cells were treated with doxycycline for 48 hrs, harvested and the lysates subjected to western blotting with phospho-specific antibodies. Increased phosphorylation of CDK1 at Y15 (Fig 2B) and PP1α at T320 (Fig 2C) was observed in the presence of wild-type BGLF4 expression.

We had previously shown that BGLF4 phosphorylation of TIP60 was a key upstream event in the BGLF4 induced activation of DNA damage signaling [6]. PP1 regulates a wide range of cellular functions including several aspects of the DDR [55]. We investigated whether there might be a linkage between PP1α and TIP60. An immunoprecipitation assay performed using V5-PP1α and Flag-TIP60 transfected cells revealed co-precipitation of PP1α with Flag–TIP60 (Fig 2D, lane 2). No PP1α was seen in the Flag immunoprecipitate from the vector control co-transfected cells (Fig 2D, lane 3). A co-precipitation assay was also performed on Flag-BGLF4 and V5-PP1α transfected cells. Co-precipitation of PP1α with BGLF4 was detected (Fig 2D, lane 1). However, when the relative amounts of Flag-BGLF4 and Flag-TIP60 present in the direct precipitates is taken into account, the association of BGLF4 with PP1α appears relatively weak compared to the PP1 and TIP60 interaction. The interaction of PP1α and TIP60 suggests that TIP60 phosphorylation may be normally regulated by PP1α. On the other hand, BGLF4 induced PP1 inhibition might also lead to p38α activation [56](Fig 2E). Activated p38α could trigger TIP60 activity by phosphorylation of T139 [57,58], which was detected with an approximately 10-fold increase in our screen (S1 Table). Therefore, BGLF4 appears to regulate multiple kinases and phosphatases to modulate the final signaling readout (Fig 2E).

### Phosphorylation motif analysis

**Fig 3:**
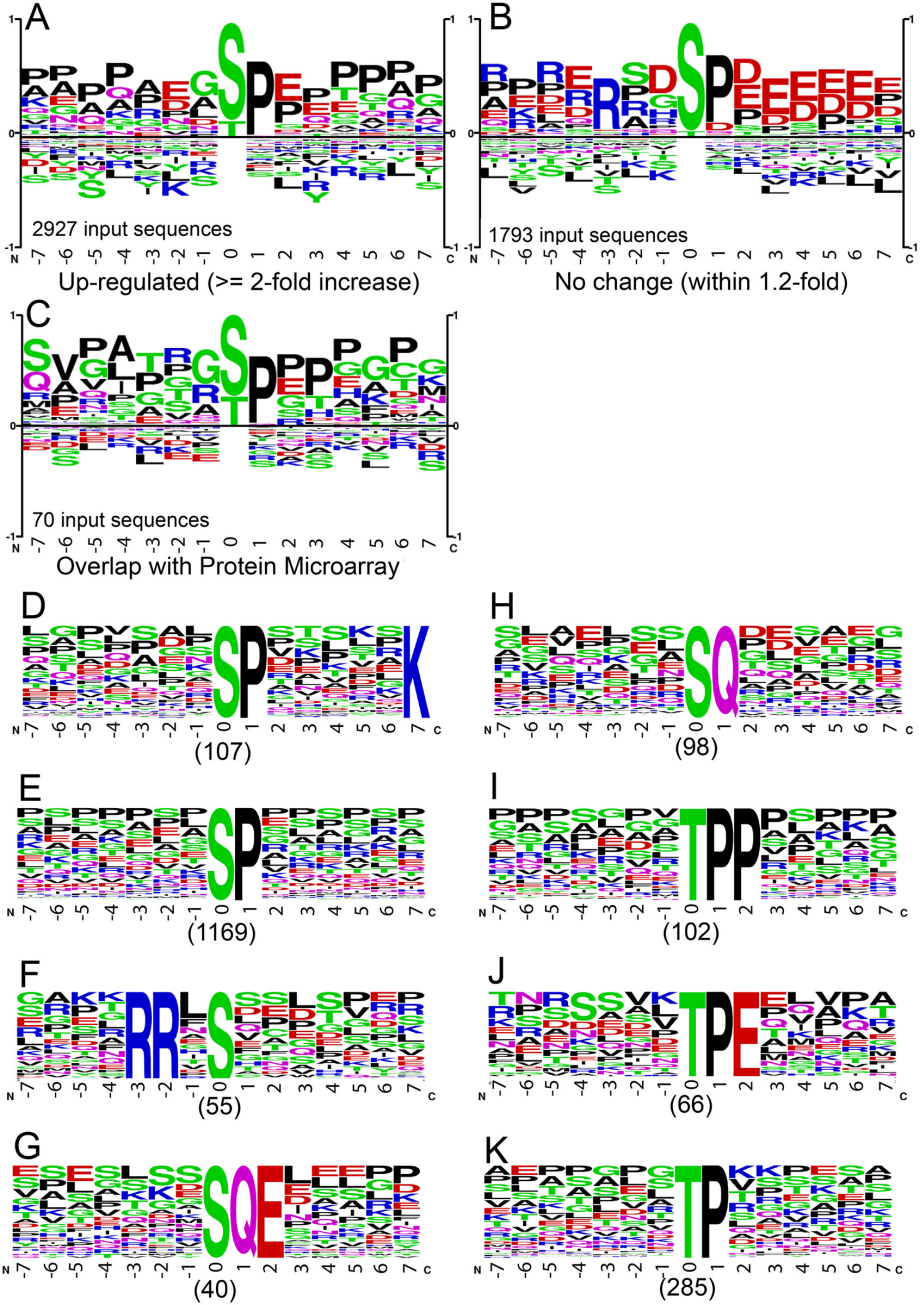
Motif analysis of phosphopeptides identified in BGLF4 expressing cells. (A-B) Dominant phosphorylation motifs showing up-regulation (A) or no change (B) in BGLF4 expressing Akata (EBV+) cells. (C) Dominant phosphorylation motif derived from hyperphosphorylated peptides extracted from the shared proteins identified by MS analysis and *in vitro* protein microarray screening. Residues above the midline were overrepresented and those below were underrepresented. (D-K) Phospho-serine (D-H)/threonine (I-K) motifs from BGLF4 up-regulated peptides (n = 2,927) were extracted and ranked using the Motif-X algorithm.

The preference for amino acids surrounding the phosphorylation site is one of the major mechanisms that contributes to kinase specificity [59]. Induction of BGLF4 would be expected to result in the phosphopeptides that were direct BGLF4 substrates being in the majority. PhosphoSitePlus (PSP) Logo Generator was used to analyze the unique phosphopeptides that showed a 2-fold or more increase in phosphorylation after BGLF4 induction. The dominant signature detected was an *S/TP motif where the *S/T is the phosphorylated serine/threonine. There were 2,927 *S/T phosphopeptides that were enriched in the -4 to -7 and +1 to +7 positions with prolines (Fig 3A). Further analysis of phospho-peptides derived from the shared targets of our MS analysis and the *in vitro* protein array screening [6] reveals a similar motif signature for BGLF4 (Fig 3C). Analysis of the 1,793 phosphopeptides that showed no change in phosphorylation after BGLF4 induction revealed that the *S/TP motif in these phosphopeptides was enriched in the +2 to +6 positions with aspartate and glutamate residues and in the -3, -5 and -7 positions with arginine residues (Fig 3B). Taken together, the phosphorylation signatures observed suggest that having proline residues surrounding the *S/TP favors BGLF4 phosphorylation while the presence of positively and negatively charged amino acids surrounding the *S/TP is unfavorable for BGLF4 phosphorylation.

Motif-X was used to further extract the individual phospho-serine and phospho-threonine motifs that were statistically enriched in the phosphopeptides that showed 2-fold or more up-regulation. The *SP motif (1,276 phosphopeptides) detected in this analysis showed either enrichment of lysine in the +7 position or no preferred amino acids in the 13 amino acids surrounding the *SP (Fig 3D and 3E). There were 453 *TP motif related sequences that showed either enrichment of proline/aspartic acid in the +2 position (Fig 3I–3J) or no preferred amino acids in the 13 amino acids surrounding the *TP (Fig 3K). These sequences may also represent BGLF4 phosphorylation sites. The data also reinforce the concept that BGLF4 has a substrate range that is expanded beyond CDK1 substrates as was previously observed in an *in vitro* analysis of EBV protein substrates. In that screen ~50% of BGLF4 substrates were not CDK1 substrates [21].

As illustrated in Fig 2A, BGLF4 expression affects the activity of cellular kinases AURKA/Aurora A, AURKB/Aurora B, ATM and ATR. The Motif-X program identified an RRx*S motif (55 phosphopeptides) (Fig 3E) and two *SQ motifs (138 phosphopeptides) (Fig 3F and 3G). RRx*S is a signature of Aurora kinases, serine/threonine kinases that regulate mitosis while *SQ is the core motif for the ATM and ATR serine/threonine DNA damage associated kinases. These results suggest that BGLF4 might simultaneously regulate the DDR and mitotic signaling through phosphorylation.

### DNA damage pathway signaling

**Fig 4:**
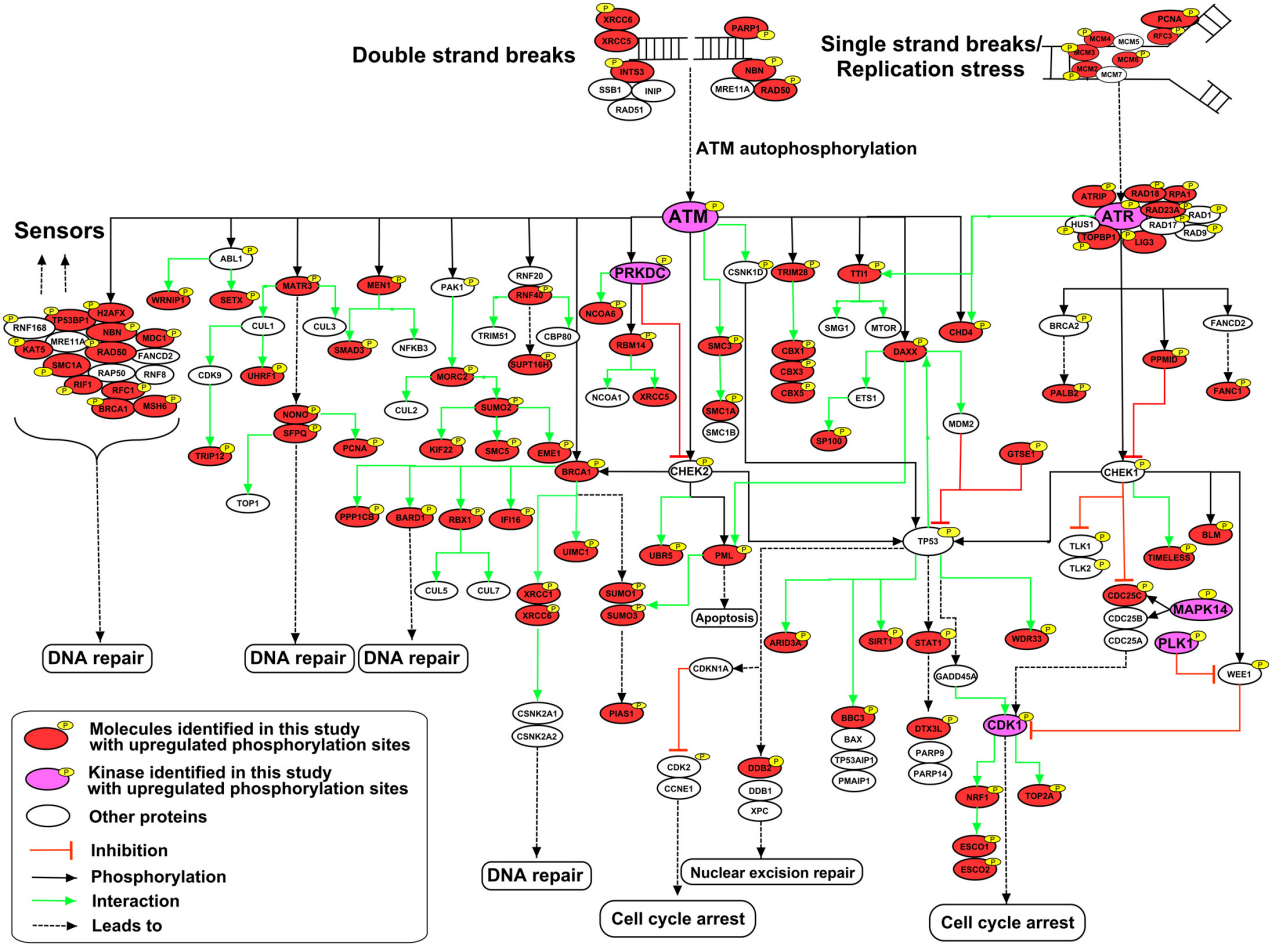
BGLF4-induced DNA damage response signaling. BGLF4 up-regulated phosphoproteins that are linked to the DNA damage response pathway were identified using the DAVID bioinformatics resource and manual literature curation. See also S3 Table.

In our previous study using human protein microarrays, we identified genes involved in the DNA damage pathway as being statistically over-represented in BGLF4 substrates [6]. In that study, 20 DNA damage pathway proteins were found to be phosphorylated *in vitro* by BGLF4. The current analysis examined the effects of BGLF4 expression in cultured cells and so detected not only direct BGLF4 phosphorylation events but also phosphorylation events mediated through the subsequent activation of downstream kinases. To obtain a more complete picture of the impact of BGLF4 on the DNA damage pathway, a list of proteins was generated that showed a 2-fold or more increase in phosphorylation after BGLF4 induction and were DNA damage-related as assessed by the David Gene Functional Classification tool (https://david.ncifcrf.gov/) plus literature curation (S3 Table). Key proteins identified are represented in the DNA damage pathway in Fig 4. The data reveal extensive phosphorylation events downstream of the ATM kinase in proteins that participate in DNA repair with a significant enrichment of proteins in the MRN (MRE11-RAD50-NBS1/NBN) complex which functions in homologous recombination repair. A more limited number of phosphorylation events were detected on proteins downstream of ATR. These results reinforce the important role of the DDR in viral replication.

### The mitotic phosphorylation network

**Fig 5:**
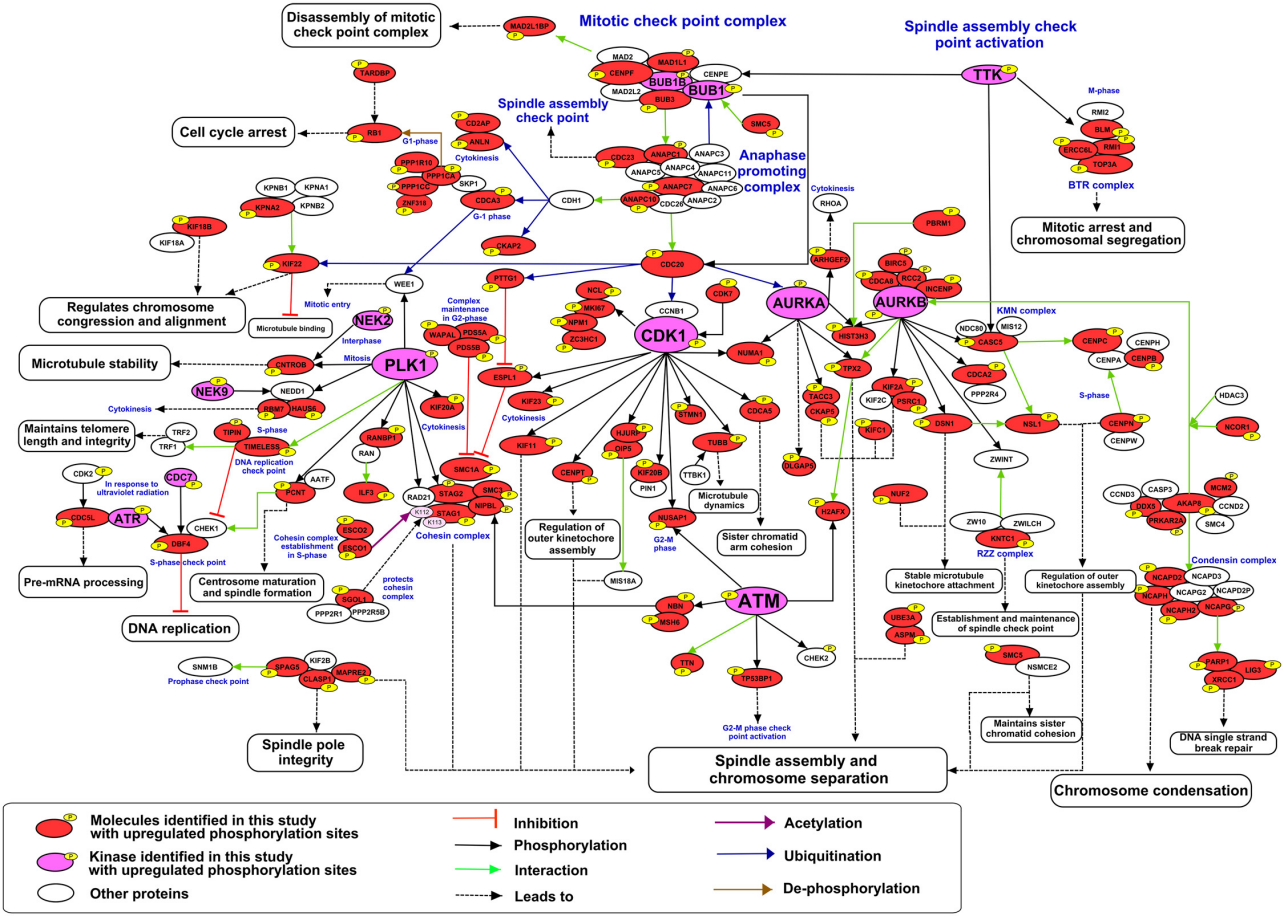
BGLF4-induced mitotic phosphorylation network. Linkage of BGLF4 up-regulated phosphoproteins to mitosis was obtained using the DAVID bioinformatics resource and manual literature curation. See also S4 Table.

We detected the phosphorylation mediated activation of several key kinases involved in the regulation of mitosis, including AURKA/Aurora A, AURKB/Aurora B, PLK1 and ATM (Fig 2A). The motif analysis also revealed a signature for the activity of Aurora kinases (Fig 3F), kinases that regulate aspects of mitosis including mitotic entry, sister chromatid cohesion and the SAC [60]. Gene Ontology (GO) analysis of the proteins whose phosphorylation was increased by 2-fold or more following BGLF4 induction yielded “Cell Cycle” and “M Phase” as two of the highest scoring groupings in the category of biological process (S4 Fig). In light of this information, we assembled a list of proteins that showed a 2-fold or more increase in phosphorylation after BGLF4 induction and were mitosis-related as assessed by the David Gene Functional Classification tool plus literature curation (S4 Table). Key proteins identified are colored in the mitosis network shown in Fig 5. CDK1/Cyclin B is a key regulator of the G2/M transition and M-phase phosphorylation. By manually examining the hyperphosphorylated sites, we extracted 233 phosphopeptides with a canonical CDK1 motif signature (S5 Table). However, in BGLF4 expressing cells, the phosphorylation activity of CDK1 appears to be suppressed and we anticipate that CDK1 kinase activity would be replaced by BGLF4. The data depicted in Fig 5 show widespread phosphorylation of substrates that lie downstream of the mitotic kinases CDK1, AURKA/Aurora A, AURKB/Aurora B and PLK1. This signaling impacts on multiple mitotic processes including spindle assembly, chromosome condensation, sister-chromatid cohesion, chromosome separation and cytokinesis [60–63].

### Activation of the spindle assembly checkpoint

In addition to the mitotic kinase signaling described above, BGLF4 impacts other aspects of the mitotic environment. Proteins showing increased phosphorylation after BGLF4 induction include components of the SAC or mitotic checkpoint complex (MCC) and the APC/C (Fig 5). APC/C has two co-activators, CDC20 and CDH1, which associate with APC/C at different stages of the cell cycle [64]. APC/C^CDC20^ is critical for orderly progression through mitosis. The mitotic checkpoint proteins, BUBR1, BUB3, and MAD2, bind to CDC20 to form the SAC which sequesters CDC20 and directly inhibits activation of APC/C^CDC20^. Inhibitory phosphorylation of CDC20 by CDK1 [65,66] and BUB1 [67] also contributes to CDC20 inhibition.

**Fig 6:**
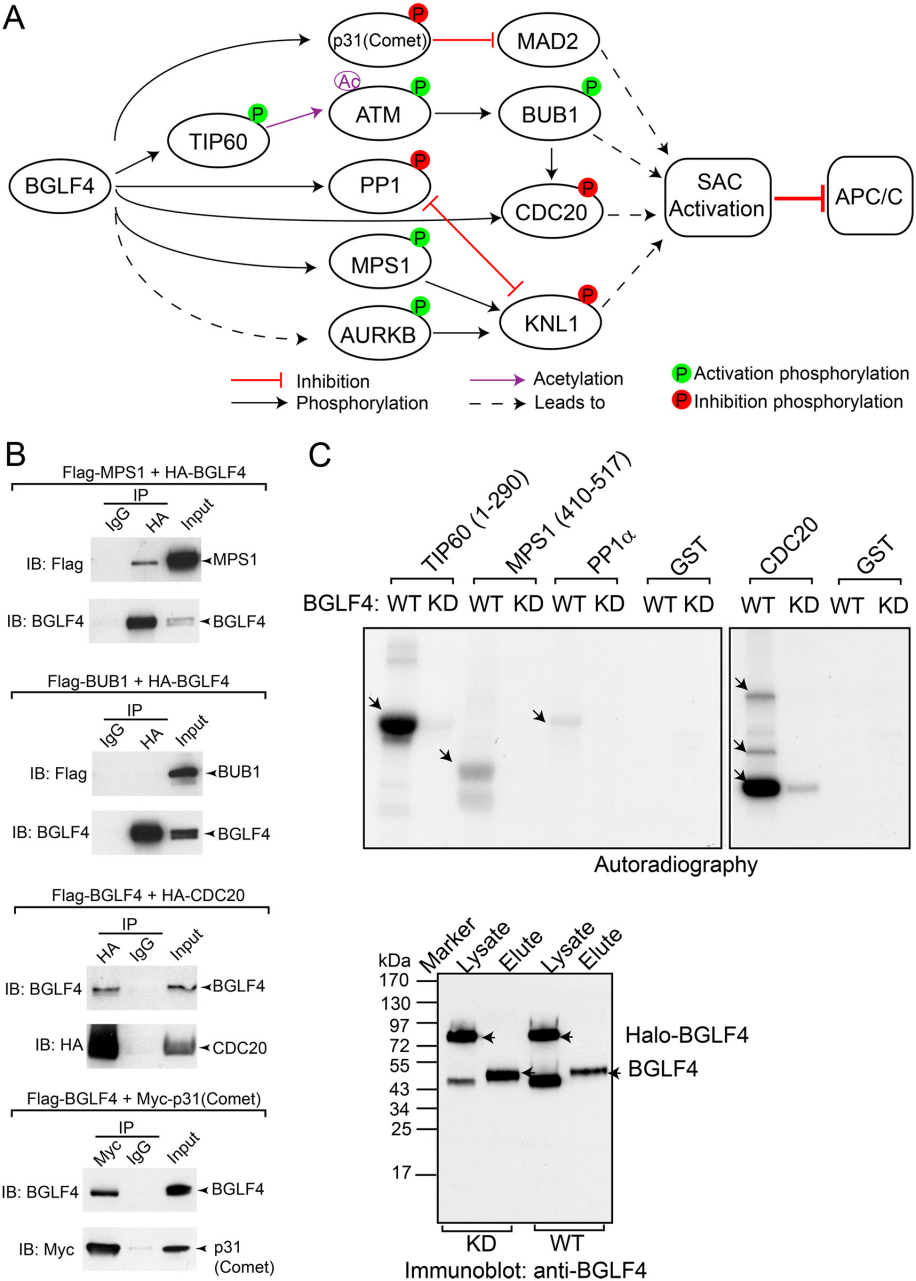
BGLF4 binds to and phosphorylates key proteins of the spindle assembly checkpoint. (A) BGLF4 induced phosphorylation of proteins involved in SAC (spindle assembly checkpoint) activation and APC/C (anaphase promoting complex /cyclosome) inhibition. P: phosphorylation; Ac: acetylation. (B) Western blot analysis showing co-precipitation of BGLF4 with MPS1, CDC20 and p31^Comet^ but not BUB1. BGLF4 was co-transfected with MPS1, BUB1, CDC20 or p31^Comet^ in 293T cells as indicated. Cell lysates were immunoprecipitated with anti-HA antibody, anti-Myc or IgG control followed by immunoblotting with anti-BGLF4, anti-HA, anti-Flag or anti-Myc antibodies as indicated. (C) BGLF4 phosphorylates TIP60, MPS1, PP1α and CDC20. (Upper) Purified wild-type (WT) and kinase-dead (KD) BGLF4 were mixed with GST-TIP60 (aa 1–290), GST-MPS1 (a.a. 410–517), GST-PP1α, GST-CDC20 and GST. Autoradiography showing only WT BGLF4 phosphorylates TIP60, MPS1, PP1α and CDC20. GST-TIP60 (a.a. 1–290) and GST were included as positive and negative controls respectively. Arrows indicate the positions of the phosphorylated substrates. (Lower) Immunoblot showing purified WT and KD BGLF4 proteins (Elute) used in the assay. Arrows indicate the positions of purified untagged BGLF4 (Elute) and Halo-BGLF4 proteins in the total cell lysate (Lysate). See also S5 Fig.

Examination of the proteins showing differential phosphorylation after BGLF4 induction revealed multiple phosphorylation events that could impact on APC/C ^CDC20^ activity (Fig 6A). MAD2L1BP (p31^Comet^) binds to Mad2, preventing Mad2 activation and silencing the SAC [68]. Phosphorylation of p31^Comet^ at S102 reduces the affinity of p31^Comet^ for Mad2 and promotes SAC activity [69]. In our analysis we observed an increase in the phosphorylation of p31^Comet^ at S102 (S134 in the isoform in our analysis) (S1 Table). We also detected a 5-fold increase in the phosphorylation of CDC20 at T70. Phosphorylation at this conserved site of *Xenopus* CDC20 correlates with a lack of association of CDC20 with APC/C [66]. Phosphorylation of MPS1/TTK at S436 has been linked to its activation [70]. Activated MPS1 can trigger the phosphorylation of kinetochore protein KNL1 to initiate SAC activation [71]. We detected an approximately 3-fold increase of MPS1 phosphorylation at this same kinase activation site (Fig 2A). Further, we detected a dramatic increase in BUB1 phosphorylation at S314 (Figs 2A and S3), a site that is phosphorylated by ATM and is essential for BUB1 kinase activity and for spindle checkpoint activation [72]. Active BUB1 also phosphorylates CDC20 and inhibits the ubiquitin ligase activity of APC/C^CDC20^ catalytically [67]. To test whether BGLF4 interacts with key mitotic proteins in cells, we performed a co-immunoprecipitation assay of transfected BGLF4 with MPS1, BUB1, CDC20 or p31^Comet^. Interestingly, we found that BGLF4 interacts with MPS1, CDC20 and p31^Comet^ but not BUB1 (Fig 6B), suggesting that BGLF4 may phosphorylate MPS1, CDC20 and p31^Comet^ directly and regulate BUB1 phosphorylation through ATM activation. To test whether BGLF4 could directly phosphorylate the proteins related to SAC activation. We performed an *in vitro* kinase assay using purified BGLF4 and GST-tagged MPS1, PP1α and CDC20. Because MPS1 itself is a protein kinase, we used only a segment of MPS1 (aa 410–517) that contains a known phosphosite (S436) in the kinase assay. As shown in Fig 6C, WT BGLF4 but not KD mutant BGLF4, phosphorylated the positive control TIP60 (a.a. 1–290) as well as MPS1 (a.a. 410–517), PP1α and CDC20. In the case of CDC20, we detected three phosphorylation bands corresponding to bands for GST tagged CDC20 and two N-terminal fragments detected by immunoblot using an anti-N-terminal CDC20 antibody (S5 Fig). These results are consistent with the hyperphosphophorylation of the N-terminal CDC20 (S41 and T70) seen in our MS analysis (S1 Table). Taken together, our results demonstrate that BGLF4 binds to and directly phosphorylates key cellular proteins to activate the SAC and block APC/C activity (Fig 6A).

**Fig 7:**
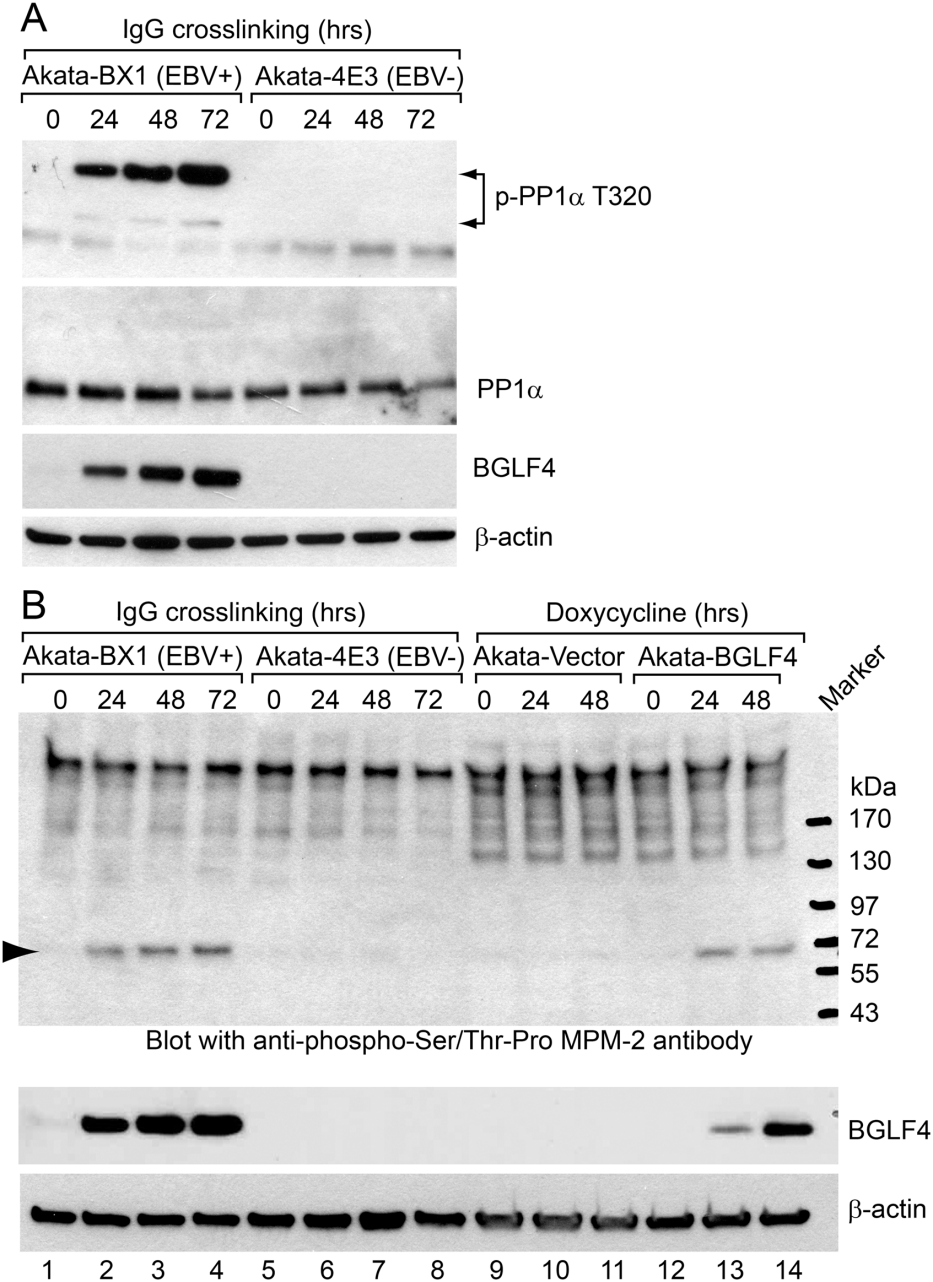
EBV lytic reactivation triggers the phosphorylation of PP1α and a mitosis-related protein. (A) EBV reactivation triggers the phosphorylation of PP1α. Western blot analysis of cell extracts from Akata-BX1 (EBV+) and Akata-4E3 (EBV-) cells using anti-phospho-PP1α T320, anti-PP1α, anti-BGLF4 and anti-β-actin antibodies as indicated. The cells were untreated (0 hr) or treated with IgG (1:200) for 24, 48 and 72 hrs as indicated. Arrow heads indicate the positions of phospho-PP1α T320. (B) EBV reactivation and BGLF4 induction trigger the phosphorylation of a mitosis-related protein. Western blot analysis of cell extracts from Akata-BX1 (EBV+) and Akata-4E3 (EBV-) cells using anti-phospho-Ser/Thr-Pro MPM2 antibody, anti-BGLF4 and anti-β-actin antibodies as indicated. The cells were untreated (0 hr) or treated with IgG or Doxycycline as indicated. Arrow head indicates the position of a phosphorylated mitotic protein.

We tested whether BGLF4 induced phosphorylation was also seen during the course of EBV lytic reactivation using phospho-specific antibodies. As shown in Fig 7A, the phosphorylation of PP1α on T320 was dramatically increased in the EBV+ cells but not the EBV- cells. In addition to PP1α, we also detected the hyperphosphorylation of a mitosis-related protein in both EBV replicating cells and BGLF4-expressing cells (Fig 7B). The phosphorylation signals detected correlate well with BGLF4 expression (Fig 7B, lanes 2–4 and 13–14).

**Fig 8:**
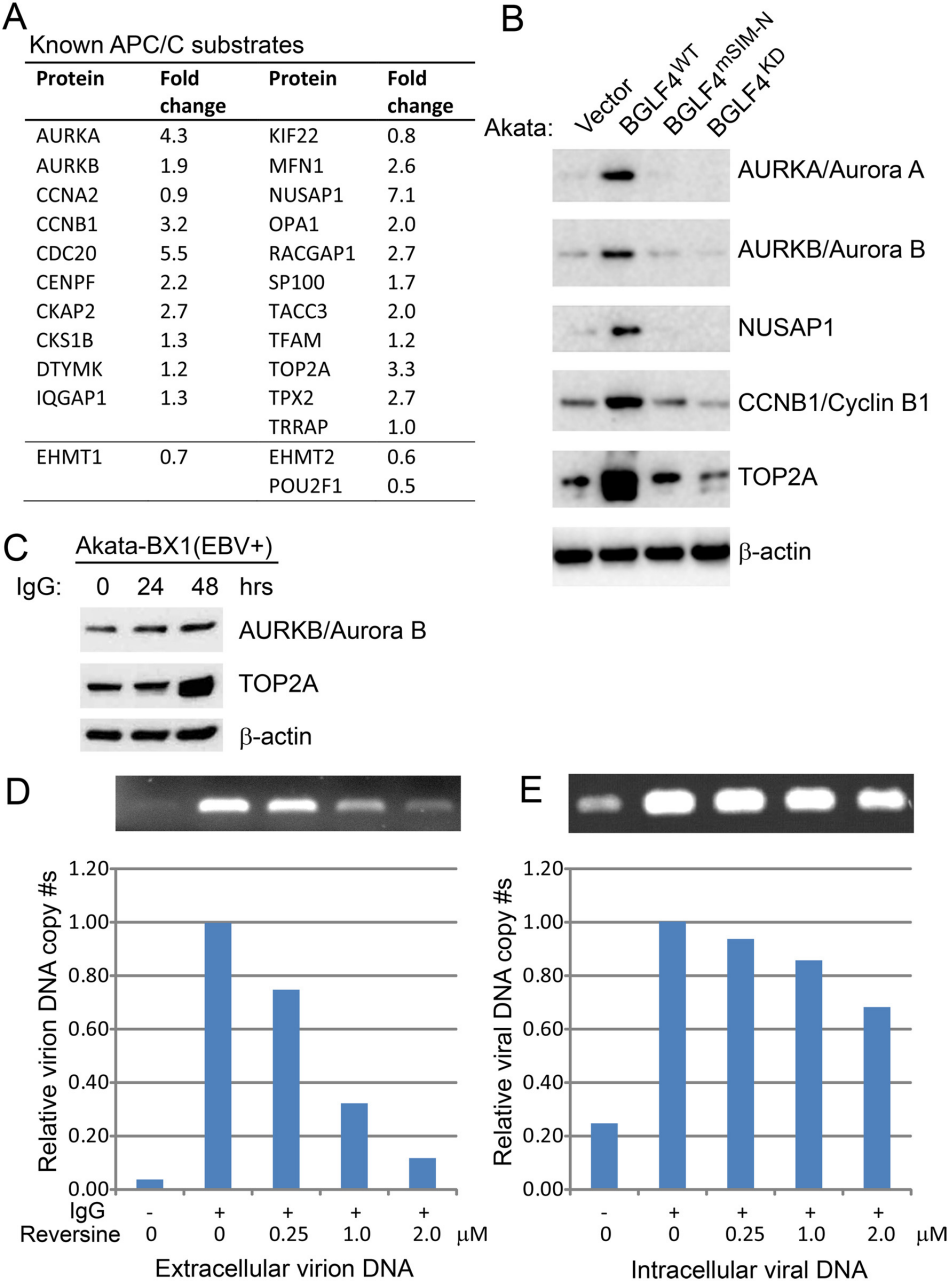
Regulation of known APC/C substrates by BGLF4. (A) Fold change in protein level of APC/C substrates as measured by MS. See also S2 Table. (B) Western blot validation of BGLF4 mediated up-regulation of individual APC/C substrates in Dox inducible Akata (EBV+) cells expressing wild-type BGLF4, but not SUMO binding-deficient and kinase-dead mutants (BGLF4^mSIM-N^ and BGLF4^KD^). Immunoblot analysis of the same cell lysates from Fig 2C using antibodies as indicated. (C) EBV replication induces the accumulation of TOP2A and Aurora B. Western blot analysis of cell extracts from Akata-BX1 (EBV+) treated as indicated using anti-Aurora B, anti-TOP2A and anti-β-actin antibodies as indicated. (D) and (E) SAC inhibition suppresses production of extracellular EBV virus. Supernatant virion DNA (D) and cell associated viral DNA (E) from Akata (EBV+) cells treated as indicated was determined by PCR. The experiments were carried out in three biological replicates with similar results and the representative results are presented. Relative PCR product intensity was quantified by ImageJ software.

APC/C is a multi-protein complex that has ubiquitin ligase activity. Substrates contain recognition elements such as the D-box, KEN-box and A-box [73] and multiple substrates and potential substrates have been identified [40,74–76]. Among the proteins detected in our MS analysis were 21 known APC/C substrates whose protein levels were either unchanged or increased (0.8- to 7.1-fold) following BGLF4 expression (Fig 8A). In contrast, only 3 known APC/C substrates had decreased protein levels after BGLF4 expression (0.5 to 0.7-fold change). Consistent with our MS data, increased protein levels of Aurora A, Aurora B, NUSAP1, Cyclin B1, and TOP2A were confirmed by western blot analysis upon wild-type BGLF4 induction (Fig 8B). In contrast, there was no protein level increase with the induction of SUMO-binding deficient or kinase dead BGLF4 mutants (Fig 8B), indicating that both SUMO binding and kinase activity are required for the BGLF4 induced increase in these APC/C substrates. In addition, we also confirmed the accumulation of Aurora B and TOP2A in lytically induced EBV+ cells (Fig 8C). These observations suggest that, by activating the SAC and blocking APC/C activity, BGLF4 may regulate the stability of proteins critical for viral replication.

To evaluate the contribution of SAC activation to EBV replication, we tested the effects of the inhibitor reversine which has been shown to inhibit the SAC [77]. Interestingly, we found that the amount of EBV virus released to the medium was dramatically reduced by treatment with reversine at doses of minimal toxicity. In contrast, the intracellular viral DNA level was less affected by reversine, suggesting that SAC activation has a role in the viral life cycle subsequent to the DNA replication step (Fig 8D and 8E).

### The nuclear pore complex

**Fig 9:**
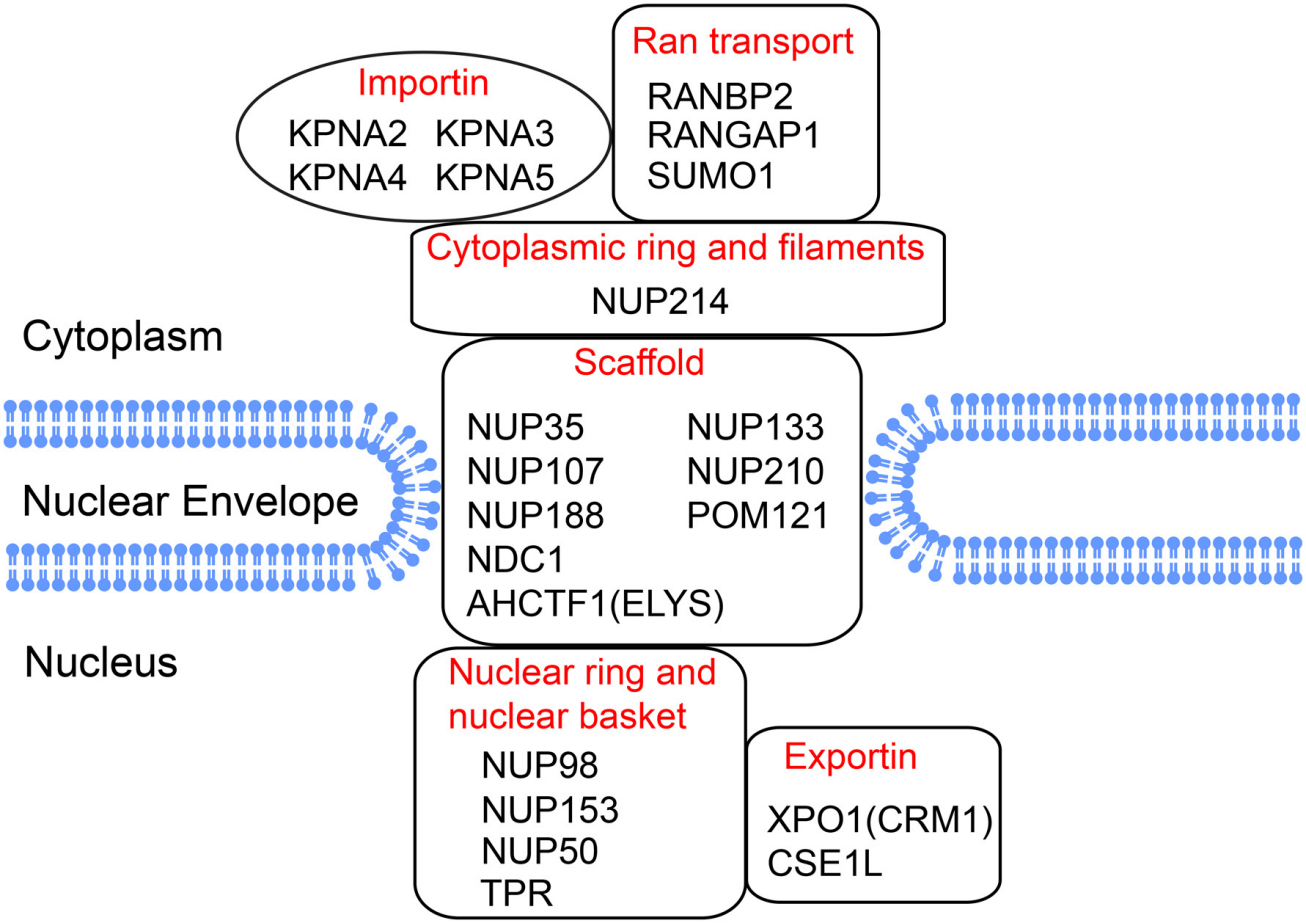
BGLF4-induced phosphorylation of nuclear pore associated proteins. Localization of BGLF4 up-regulated phosphoproteins associated with the nuclear pore as identified using the DAVID bioinformatics resource and manual literature curation. See also S6 Table.

Nuclear envelope breakdown takes place at the transition into prometaphase and is a critical requirement for mitotic entry. Hyperphosphorylation of nuclear pore proteins takes place during mitosis and we noted increased phosphorylation of multiple nuclear pore proteins after BGLF4 expression (Fig 9 and S6 Table). Phosphorylation of the nucleoporin NUP98 has been identified as being particularly important for nuclear pore disassembly [78]. NUP98 is phosphorylated by the kinases Nek6 and CDK1. We detected 11 hyperphosphorylation events on NUP98 after BGLF4 expression, including increased phosphorylation of one of two Nek6 sites (S608) and three of five CDK1 sites (T546, S612 and S623) (S6 Table). BGLF4 has previously been shown to interact with the nuclear pore proteins NUP62 and NUP153 and to increase the permeability of the nuclear pore for large proteins [27]. Here we detected increased phosphorylation on 6 sites of NUP153 that may be potentially phosphorylated by BGLF4. The increased phosphorylation of importins (KPNA2/3/4/5) and RAGAP1-SUMO1 also suggests that BGLF4 might regulate the relocalization of importins and RAGAP1-SUMO1 through phosphorylation and therefore enhance nuclear transport of viral proteins [27]. This is supported by the observation that BGLF4 increased the expression of KPNA2 and KPNA4 at the protein level in the nuclear fraction by 2- to 3-fold in our proteomic analysis (S2 Table).

## Discussion

We utilized SILAC-based quantitative proteomics approaches to globally identify the downstream phosphorylation events regulated by the EBV protein kinase BGLF4. Our study revealed that BGLF4 initiates widespread changes in protein phosphorylation to impact multiple cellular processes, in particular, the DDR, mitosis, cell cycle and nuclear transport. This study provides the most comprehensive survey of quantified phosphorylation events triggered by a conserved herpesvirus protein kinase.

We provide insight into the substrate motifs phosphorylated after expression of the EBV conserved herpesvirus protein kinase (Fig 3). The phosphorylation triggered by BGLF4 was partially mediated by BGLF4 modulation of cellular kinase and phosphatase activity. We detected activation phosphorylation events on ATM, ATR, Aurora A, BUB1, PLK1 and p38α and inhibitory phosphorylation events on phosphatases CDC25C and PP1 (Fig 2A). We also observed increased protein levels of Aurora kinases A and B upon BGLF4 induction (Fig 8A and 8B). Consistent with these observations, motifs resembling those of ATM/ATR and Aurora A/B were extracted from the BGLF4 up-regulated phosphoproteins (Fig 3F–3H). Although BGLF4 partially mimics cellular CDK activity [24], the large number of proline-directed phosphopeptides without CDK1 motif features reinforces the previous observation [21] that BGLF4 has a broader substrate specificity than CDK1 (Fig 3). A recent study on phosphorylation induced by murine γ-herpesvirus 68 (MHV68) infection also found a CDK motif signature. In that case the signature was generated predominantly by the D-type cyclin ortholog ORF72 rather than the BGLF4 ortholog ORF36 [79]. D-type cyclins are encoded by gamma 2 herpesviruses (rhadinoviruses) but not the gamma 1 herpesviruses (lymphocryptoviruses) of which EBV is a member. These observations suggest that EBV BGLF4 and MHV68 ORF36 protein kinases may have evolved differing substrate specificities and emphasize the potential for selective activation of CDK1-like substrates during herpesvirus replication.

We provide the largest dataset on the DDR pathway regulated by the conserved herpesvirus protein kinases. Previously, we found that BGLF4 regulates the host DDR through the TIP60/ATM pathway [6]. The ATM dependent DDR plays a critical role in EBV lytic reactivation in both B cells and epithelial cells [6,80]. The current study significantly expands the list of DDR proteins regulated by BGLF4 (Fig 4 and S3 Table). In addition to manipulation of ATM signaling, BGLF4 also positively regulated ATR. Not all the hyperphosphorylated sites in the DDR pathway contain an ATM/ATR motif (S3 Table), suggesting that BGLF4 may directly phosphorylate certain DDR proteins or modulate other cellular kinase or phosphatase activity to impact the final signaling readout. Our study suggests an extensive participation of DDR proteins in viral life cycle. The importance of our finding is further supported by the fact that DDR signaling also plays a key role in the replication of a variety of viruses [81–84] and that viral latency proteins tend to block the DDR to facilitate latency establishment [6,85–87].

We now show that BGLF4 also increases the phosphorylation of a large number of proteins involved in the mitotic phase of the cell cycle (Fig 5 and S4 Table). EBV genome amplification arrests the cell in early S phase to create a pseudo-S phase environment [88] and BGLF4 is known to contribute to the pseudo-S phase environment through Rb and p27 phosphorylation [23,24]. Virus-induced pseudo-mitosis is also observed in cells replicating human cytomegalovirus (HCMV) [89]. The widespread regulation of mitotic signaling by BGLF4 seen in our study suggests that the mitosis-like environment is intimately involved in EBV replication and maturation.

A new insight into BGLF4 regulated mitotic signaling comes from the evidence of SAC activation through protein phosphorylation (Figs 6 and 7). The key regulators of the SAC, CDC20, BUB1 and MPS1, are phosphorylated either directly by BGLF4 or via BGLF4 mediated activation of ATM. SAC activation plays a critical role in inhibiting APC/C ubiquitin ligase activity. The central role of the APC/C complex in regulating the cell cycle has led to the APC/C being targeted by viruses to benefit their life cycles [90,91]. We found that the protein level of multiple known APC/C substrates is increased upon BGLF4 induction, indicating inhibition of APC/C activity by BGLF4 (Fig 8A and 8B). Furthermore, we also demonstrated that the BGLF4 induced accumulation of TOP2A and Aurora B can be observed in lytically induced Akata (EBV+) cells (Fig 8C). The APC/C target TOP2A has previously been shown to be important for both EBV and KSHV replication [92–95]. During HCMV infection, the viral protein kinase UL97, together with UL21a, down-regulates APC/C activity by targeting its subunits [96]. We anticipate that, during EBV lytic replication, other viral proteins may also co-ordinate with BGLF4 to selectively regulate the SAC readout and the ultimate stability of individual APC/C components. Interestingly, our study indicates that SUMO binding and kinase activity are critical for BGLF4 induced APC/C inhibition (Fig 8B) as well as the phosphorylation of PP1 (Fig 2C), TIP60, ATM, KAP1 and H2AX [29]. BGLF4 induction of the DDR is driven by BGLF4/TIP60/ATM signaling [6]. There is cross-talk between the DDR and the SAC that is mediated by ATM [97–99]. The effectiveness of ATM inhibitors in blocking EBV replication [6] may therefore be partially explained by the participation of ATM in two checkpoints whose modulation is critical for EBV replication. Importantly, we have demonstrated that the amount of extracellular EBV virus released is significantly suppressed by a small molecule inhibitor reversine (Fig 8D and 8E). Reversine is reported to block SAC activation by inhibiting the mitotic kinase activity of MPS1/TTK and Aurora A and B. Recently reversine was also shown to reverse the aberrant prolonged mitosis duration triggered by human papillomavirus (HPV) E6/E7 [77,100,101]. Taken together, our observations suggest that the mitotic signaling triggered by BGLF4 plays an important role in the production of mature extracellular virus.

Lytic viral replication is also associated with inhibition of APC/C activity in HCMV and high risk HPV infections [90,102–104] although in these cases the inhibition occurred through sequestration or degradation of APC/C subunits. More frequently described are examples of viral latency proteins that promote APC/C activity. HPV16 E6 and E7 up-regulate CDC20 and Ubch10 and promote APC/C activity [105] while EBV EBNA2 induces MAD2 degradation and possibly activation of APC/C [106] and human T cell lymphotropic virus type 1 (HTLV-1) tax promotes APC/C activity through binding to CDC20 and APC3 [107]. Hepatitis B virus (HBV) X protein activates APC/C through inhibiting the association between BubR1 and CDC20 [108] and simian virus 40 (SV40) large T antigen disrupts the MCC/SAC complex and promotes APC/C activity [109]. Thus the latency and lytic phases of the virus life cycle are diametrically opposed in their interactions with the SAC.

We also provide evidence that the nuclear pore complex is extensively regulated by BGLF4 phosphorylation. BGLF4 has been shown to target the nuclear pore complex through interaction with nucleoporins and BGLF4 mediated phosphorylation of NUP62 and NUP153 has been implicated in the nuclear transport of viral proteins [27,110]. We noted 6 phosphorylation sites on NUP153 after BGLF4 induction (S6 Table). In addition, our current study identified phosphorylation sites on other proteins involved in the nuclear pore complex including NUP35, NUP50, NUP98, NUP107, NUP133, NUP188, NUP210, NUP214, ELYS, NDC1, POM121, XPO1, CSNE1L and TPR (Fig 9 and S6 Table). Phosphorylation of these proteins may play a role in BGLF4 induced nuclear pore disassembly [78]. Interestingly, a recent study also detected the phosphorylation of NUP98 and NUP153 by the HCMV protein kinase UL97 [111], indicating the conserved role of these viral kinases in targeting the nuclear pore complex during viral replication. The nuclear pore complex has also been implicated in regulation of the SAC [112]. The Mad1-Mad2 complex is a key mediator of the SAC. In interphase, Mad1-Mad2 is docked at the nucleoplasmic side of the nuclear pore complex by interactions with the nuclear basket protein TPR [113,114]. Upon disassembly of the nuclear pore, the Mad1-Mad2 complex is recruited to BUB1 to allow SAC formation [115]. BGLF4 may therefore also contribute to SAC activity via nuclear pore disassembly.

In summary, our study suggests that the EBV protein kinase BGLF4 integrates multiple signaling events, including the DDR, mitotic signaling and phosphorylation of the nuclear pore complex, to induce SAC activation and APC/C inhibition. The information obtained gives valuable insight into viral manipulation of host signaling pathways that facilitate lytic replication and thus provides a foundation for the design of therapeutic strategies to limit EBV-associated disease.

## Materials and Methods

### Cell culture and reagents

The Akata (EBV+)-tet-Vector, Akata (EBV+)-tet-BGLF4, Akata (EBV+)-tet-BGLF4 (mSIM-N), Akata (EBV+)-tet-BGLF4 (KD), Akata-BX1 (EBV+), Akata (EBV+) and Akata-4E3 (EBV-) cells were grown in RPMI 1640 media supplemented with 10% FBS (Cat# 26140079, Thermo Fisher Scientific) in 5% CO_2_ at 37°C [6,29]. 293T cells were grown in DMEM media supplemented with10% FBS (Cat# 26140079, Thermo Fisher Scientific) in 5% CO_2_ at 37°C. The construction of Akata (EBV+)-tet-Vector, Akata (EBV+)-tet-BGLF4, Akata (EBV+)-tet-BGLF4 (mSIM-N), and Akata (EBV+)-tet-BGLF4 (KD) cell lines was described previously [21]. In order to label cells with stable isotopic amino acids, Akata (EBV+)-tet-BGLF4 and Akata (EBV+)-tet-Vector cells were propagated in RPMI 1640 SILAC media deficient in both L-lysine and L-arginine (Cat# 88421, Thermo Fisher Scientific) and supplemented with light lysine (^12^C_6_^14^N_2_-K) and arginine (^12^C_6_^14^N_4_-R) for light state (Cat#s L-9037 and A-8094, Sigma), and ^13^C_6_^15^N_2_-K and ^13^C_6_^15^N_4_-R for heavy state labeling (Cat#s CNLM-291-H-1 and CNLM-539-H-1, Cambridge Isotope Laboratories). Cells were cultured for at least 6 doubling times for complete incorporation. V5-PP1α plasmid was kindly provided by Christine Neuveut [116]. Flag-TIP60 and Flag-BGLF4 were described previously [6,29]. MPS1 and BUB1 were obtained from William Hahn and David Root (Addgene plasmid #s 23857 and 23612) [117] and were recloned as Flag-MPS1 and Flag-BUB1 in an SG5 expression vector. HA-cdc20 was a gift from Marc Kirschner (Addgene plasmid # 11594)[64]. pcDNA5 FRT TO myc p31^Comet^ was a gift from Stephen Taylor (Addgene plasmid # 59833) [68]. TIP60 (a.a. 1–290), MPS1 (a.a. 410–517), PP1α and CDC20 were cloned into a modified GEX-2T vector (GH413). Halo-BGLF4 (WT) and Halo-BGLF4 (KD) were constructed by cloning WT and KD BGLF4 into pHTN HaloTag CMV-neo Vector (Cat# G7721, Promega).

### Cell lysis and immunoblotting

Cells were harvested and lysed in 2x SDS-PAGE sample buffer and boiled for 5 minutes. The samples were separated on 4–20% TGX gels (Biorad), transferred to PVDF membranes, and probed with primary and horseradish peroxidase-conjugated secondary antibodies. Primary antibodies purchased from Cell Signaling Technology were: anti-phospho-CDC2/CDK1-Y15 (Cat# 9111), anti-CDC2/CDK1(Cat# 9112), anti-Phospho-PP1α-T320 (Cat# 2581), anti-PP1α (Cat# 2582), anti-γH2AX (Cat# 2595), anti-Cyclin B1 (Cat# 12231), anti-TOP2A (Cat# 12286), anti-CDC20 (Cat# 4823), Aurora kinase sampler kit (Cat# 3875) and phosphor-Ser/Thr kinase substrate antibody sampler kit (Cat# 9920). Rabbit anti-β-actin polyclonal antibody (Cat# A5441), anti-Flag (Cat# F7425), anti-NUSAP1 (Cat# SAB4502109) and mouse anti-Myc antibodies (Cat# M4439) were obtained from Sigma Aldrich. Rat anti-hemagglutinin (HA) high-affinity antibody (Cat# 11-867-431-001) was obtained from Roche. Mouse anti-V5 and anti-V5-HRP antibodies (Cat# R960-25 and R961-25) were obtained from Invitrogen. Anti-phospho-Ser/Thr-Pro MPM2 antibody (Cat# 05–368) was purchased from Millipore. Normal rat IgG (Cat# sc-2026) and mouse IgG (Cat# sc-2025) were obtained from Santa Cruz. Mouse anti-BGLF4 antibody has been described previously [118].

### Subcellular fractionation and sample preparation

Cells were the treated with doxycycline for 48 hrs and then counted twice using a hemocytometer and confirmed to be greater than 95% viable by Trypan blue exclusion. Equal numbers of cells (1x10^9^) from light and heavy conditions were mixed and cells were centrifuged at 400 x g for 5 min. Pellets were washed twice by re-suspending in 250 ml of pre-chilled Dulbecco’s PBS (Cat# 10010–049, Thermo Fisher Scientific). Nuclear fractionation was performed using nitrogen cavitation as previously described [41] with some modifications. Briefly, the combined cells were lysed in Hypotonic Lysis Buffer (20 mM HEPES pH 7.4, 10 mM KCl, 2 mM MgCl_2_ and 2mM CaCl_2_) plus protease (protease inhibitor tablet, Roche Cat# 05892791001) and phosphatase inhibitors [(2.5 mM sodium pyrophosphate (Na_4_P_2_O_7_), 1 mM sodium orthovanadate (Na_3_VO_4_), 1 mM β-glycerophosphate, 10 mM sodium fluoride (NaF)] for subcellular fractionation by nitrogen cavitation at 200 psi for 10 min on ice (4639 Cell Disruption Vessel, Parr Instrument Company). Cell disruption with intact nuclei was confirmed by trypan blue staining. The lysate was centrifuged at 1000 x g for 10 min at 4°C to collect the crude nuclei and then resuspended in 10 ml Nuclear Wash Solution (Hypotonic Lysis Buffer plus 0.25 M sucrose and protease/phosphatase inhibitors). The nuclei were harvested by centrifugation at 1000 x g for 10 min at 4°C and then were re-suspended in 10 ml freshly prepared Urea Lysis Buffer (20 mM HEPES pH 8.0, 9 M urea and protease/phosphatase inhibitors) for sonication. The nuclear lysate was centrifuged at 15,000 x g for 10 min at 18°C. The supernatant was stored in -80°C for proteomics analysis. Protein concentration (~4 mg/ml) was measured by BCA assay (Pierce, Cat# 23227).

Peptides were prepared by an in-solution tryptic digestion protocol with modifications [119]. Briefly, nuclear lysate (in Urea Lysis Buffer) was reduced with 4.5 mM dithiothreitol and alkylated with 10 mM iodoacetamide. For tryptic digestion, protein extracts were diluted in 20 mM HEPES pH 8.0 to a final concentration of 2 M urea and incubated with TPCK-treated trypsin (Cat# LS003744, Worthington Biochemical) at 25°C overnight. Protein digests were acidified by 1% v/v trifluoroacetic acid (TFA) and subjected to centrifugation at 2,000 × g at 25°C for 5 min. The supernatant of the protein digests was loaded onto a Sep-Pak C18 column (Cat# WAT051910, Waters, Columbia, MD) equilibrated with 0.1% v/v TFA. Columns were washed with 6 ml of 0.1% v/v TFA twice and peptides were eluted in 2 ml of 40% v/v acetonitrile (ACN) with 0.1% v/v TFA three times. Eluted peptides were lyophilized and subjected to high pH reversed-phase liquid chromatography (bRPLC) fractionation.

Peptides were fractionated by bRPLC as described earlier [120,121]. Briefly, 12 mg lyophilized peptide mixture was re-suspended in buffer A [10 mM triethylammonium bicarbonate (TEABC)] and fractionated by bRPLC chromatography on an Agilent 1100 LC system using a linear gradient of 8 to 60% buffer B (10 mM TEABC in 90% ACN) for 60 min at a flow rate of 1 ml per min. A total of 96 fractions were collected, concatenated to 12 fractions, and vacuum dried. For the nuclear proteomic analysis, 10% of the peptides from each fraction were used. For the phosphoproteomic analysis, the remaining 90% of the peptides were subjected to TiO_2_-based phosphopeptide enrichment using 5 um titansphere beads (Cat# 5020–75000, GL Sciences, Japan). Peptides were mixed with beads in a 1:1 ratio and incubated at RT for 30 minutes. Peptides were then washed with 80% ACN in 3% TFA, eluted using a 4% ammonia solution and immediately neutralized with 4% TFA. Eluted peptides were vacuum dried, re-suspended in 30 μL 0.1% TFA, and desalted using C_18_ StageTips. The eluted peptides were subjected to LC-MS/MS analysis.

### LC-MS/MS analysis

The total nuclear peptides were analyzed on an LTQ-Orbitrap Velos mass spectrometer interfaced with Easy-nLC II nanoflow LC system (Thermo Scientific). The mass spectrometer was operated in the data dependent mode, precursor and product ions were selected and measured using Orbitrap mass analyzer. The peptides were loaded onto a pre-column (75 μm x 2 cm, Magic C18 AQ 5 μm, 120 Å) and resolved on an analytical column (75 μm x 20 cm, Magic C18 AQ 3 μm, 120 Å) in 0.1% v/v formic acid and eluted using an ACN gradient (3–35% v/v) containing 0.1% v/v formic acid for 95 minutes and a total run time of 120 minutes. The settings were: a) Precursor scans acquired (FTMS) from 350–1,800 m/z at 60,000 resolution; and b) MS2 scans acquired (FTMS)- fragmented using higher energy collisional dissociation (HCD) fragmentation of the 10 most intense ions (isolation width: 1.90 m/z; normalized collision energy: 35%; activation time = 0.1 ms) at 30,000 resolution.

The enriched phosphopeptides were analyzed on an LTQ-Orbitrap Elite mass spectrometer interfaced with Easy-nLC II nanoflow LC system (Thermo Scientific). The peptide digests were reconstituted in 0.1% formic acid and loaded onto a trap column (75 μm x 2 cm) packed in-house with Magic C18 AQ (Michrom Bioresources, Inc., Auburn, CA, USA). Peptides were resolved on an analytical column (75 μm x 50 cm) at a flow rate of 300 nL/min using a linear gradient of 10–35% solvent B (0.1% formic acid in 95% ACN) over 85 min. The total run time including sample loading and column reconditioning was 120 min. Data dependent acquisition with full scans in 350–1700 m/z range was carried out using an Orbitrap mass analyzer at a mass resolution of 120,000 at 400 m/z. The fifteen most intense precursor ions from a survey scan were selected for MS/MS fragmentation using higher energy collisional associated dissociation (HCD) fragmentation with 32% normalized collision energy and detected at a mass resolution of 30,000 at 400 m/z. Automatic gain control (AGC) target was set to 1x10^6^ for MS and 5x10^4^ ions for MS/MS with a maximum accumulation time of 200 ms. Dynamic exclusion was set for 30 seconds with a 10 ppm mass window. Internal calibration was carried out using lock mass option (m/z 445.1200025) using ambient air.

### MS data analysis

The MS derived data were screened using MASCOT (Version 2.2.0) and SEQUEST search algorithms against a human RefSeq database (version 70) plus EBV database from the Akata strain [122] using Proteome Discoverer 2.0 (Thermo Scientific). The search parameters for both algorithms included: carbamidomethylation of cysteine residues as a fixed modification and protein N-terminal acetylation, oxidation at methionine, phosphorylation at serine, threonine and tyrosine and SILAC labeling ^13^C_6_^15^N_2_-K and ^13^C_6_^15^N_4_-R as variable modifications. MS/MS spectra were searched with a precursor mass tolerance of 20 ppm and fragment mass tolerance of 0.1 Da. Trypsin was specified as the protease and a maximum of two missed cleavages were allowed. The data were screened against a target decoy database and the false discovery rate was set to 1% at the peptide level. The protein and peptide data were extracted using top one peptide rank and high peptide confidence filters. The false discovery rate (FDR) was calculated by enabling the peptide sequence analysis using a decoy database and a cut-off of 1% peptide and protein level FDR used for identifications. The SILAC ratio for each phosphopeptide-spectrum match (phosphoPSM) was calculated by the quantitation node and the probability of phosphorylation for each Ser/Thr/Tyr site on each peptide was calculated by the ptmRS node in the Proteome Discoverer and phosphosite probabilities greater that 75% were extracted for further analyses. Peptides with ratios greater than 2.0-fold were considered as regulated and used for further analysis.

The MS proteomics data have been deposited to the ProteomeXchange Consortium (http://proteomecentral.proteomexchange.org) via the PRIDE partner repository with the dataset identifier PXD002411.

### Motif analysis

The surrounding sequence (7 amino acid residues on either side) for each identified phosphorylation site was extracted from the RefSeq database (version 70). For phosphorylation sites that were located at the N-or C-termini, the surrounding sequence could not be extended in this fashion and they were excluded from further motif analysis. The Motif-X algorithm (http://motif-x.med.harvard.edu/) was used to extract motifs. The significance threshold was set to p<1 x 10^−10^. The minimum occurrence of the motif was set to 30 for pSer/pThr peptides against an IPI Human proteome background. Motif logos were generated by WebLogo (http://weblogo.berkeley.edu/). PhosphoSitePlus (PSP) Logo Generator was used to plot the frequency of amino acid residues on each position (http://www.phosphosite.org/sequenceLogoAction.do). Residues above the midline were over-represented and those below were under-represented.

### Bioinformatics analysis

The occurrence of each Gene Ontology (GO) was obtained from the DAVID Bioinformatics Resource (https://david.ncifcrf.gov/). The DNA damage response pathway and mitosis maps were constructed by manual curation of the literature combined with the information from the DAVID Bioinformatics Resource.

### Protein expression and purification

GST-tagged proteins were expressed and purified as described previously [29,123]. Briefly, *Escherichia coli* BL21 cells were transformed with expression vectors [TIP60 (a.a. 1–290), MPS1 (a.a. 410–517), PP1α and CDC20] and then cultured in LB medium at 37°C until the A_600_ reached 0.6. The bacteria were induced by adding 0.1 mM IPTG (isopropyl-β-D-thiogalactopyranoside) at 16°C for 12 to 16 hrs. Bacteria were harvested and then lysed by sonication. GST fusion proteins were purified by affinity chromatography using Glutathione-Sepharose 4B (Cat# 17-0756-01, GE Healthcare) according to the manufacturer’s instructions.

Wild-type (WT) and kinase-dead (KD) BGLF4 were purified from 239T cells using the Halotag Mammalian Protein Purification System (Cat# G6790, Promega) according the manufacturer’s instructions. Briefly, Halo-tagged WT and KD BGLF4 were transfected into Hela cells. Two T175 flasks of transfected cells were harvested 48 hrs post-transfection at 100% confluence and lysed with 25 ml HaloTag Protein Purification Buffer (50 mM HEPES pH7.5, 150 mM NaCl, 1mM DTT, 1mM EDTA and 0.05% IGEPAL CA-630) with Protease Inhibitor Cocktail. WT and KD Halo-BGLF4 were enriched using the Halo-tag resin and BGLF4 was eluted from the resin by washing 3 times with 0.5 ml HaloTag Protein Purification Buffer containing 20 ul Halo-TEV protease.

### *In vitro* kinase assay

Purified GST-Tip60 (a.a. 1–290), GST-PP1α, GST-MPS1 (a.a. 410–517) and GST-CDC20 on beads were washed twice with Kinase Buffer (20 mM MOPS pH 7.2, 25 mM β-glycerophosphate, 5 mM EGTA, 1 mM Na_3_VO_4_ and 1 mM dithiothreitol). Each sample was incubated in 40 ul Kinase Buffer containing 0.1 (v/v) magnesium-ATP cocktail buffer (Cat# 20–113; Upstate), 0.2 μCi of [γ-^32^P]ATP (Cat# Blue5027 PerkinElmer) and 6 μl of WT or KD BGLF4 for 30 min at 30°C with vortexing every 2 to 3 minutes. Finally, reaction mixtures were washed twice with ice-cold Kinase Buffer and separated by gel electrophoresis. Radiolabeled proteins were detected by autoradiography.

### Reversine treatment and EBV DNA detection

Akata (EBV+) cells were untreated or treated with IgG (1:200, Cat# 55087, MP Biomedicals) for 3 hrs and then reversine (Cat# R3904, Sigma) was added for 5 days. To measure EBV virus release, virion-associated DNA in the culture supernatant was determined by PCR analysis using BALF5 primers as previously described [29]. The supernatant (180 μl) was treated with 4 μl RQ1 DNase (Cat# M6101, Promega) for 1 h at 37°C, and reactions were stopped by adding 20 μl of Stop Buffer and incubation at 65°C for 10 min. Then 12.5 μl Proteinase K (20 mg/ml; Cat# 4333793, Invitrogen) and 25 μl 10% (w/v) SDS were added into the reaction mixtures which were incubated for 1 h at 65°C. DNA was purified by twice phenol-chloroform extraction followed by isopropanol/sodium acetate precipitation. To measure cell associated viral DNA, total genomic DNA was extracted using the Genomic DNA Purification Kit (Cat# A1120, Promega) and relative viral DNA content was determined by PCR analysis using BALF5 primers.

## Supporting Information

S1 Fig Western blot analysis of phosphorylated substrates at different time points after BGLF4 induction by doxycycline (Dox). The motif specific antibodies recognize CDK or PKA substrates.
(TIF)

S2 Fig Scatter plot of log_2_ transformed phosphopeptide ratios (BGLF4 vs Vector) showing good correlation between two biological replicates (Pearson coefficient correlation R = 0.95). (TIF)

S3 Fig Representative MS spectra of phosphopeptides. MS spectra showing the changes in the relative abundance of phosphopeptides [light (BGLF4) vs heavy (Vector)]. Phosphosites are labeled as lowercases.
(TIF)

S4 Fig Enriched biological processes revealed by DAVID GO analysis (GOTERM_BP_FAT). (TIF)

S5 Fig (A) Coomassie Brilliant Blue (CBB) staining showing the GST-tagged proteins and GST used in the assay. Arrows indicate the positions of GST and GST tagged proteins. (B) Immunoblot of purified GST-CDC20 reacted with anti-CDC20 (N-terminal) antibody. Arrows indicate the positions of GST-CDC20 and two major GST tagged N-terminal fragments of CDC20.
(TIF)

S1 Table A complete list of phosphosites quantified. (XLSX)

S2 Table A complete list of proteins identified. (XLSX)

S3 Table A list of BGLF4-regulated phosphoproteins involved in the DNA damage response pathway. (XLSX)

S4 Table A list of BGLF4-regulated phosphoproteins involved in the mitotic phase of the cell cycle. (XLSX)

S5 Table A list BGLF4-regulated phosphopeptides with canonical CDK1 motif. (XLSX)

S6 Table A list of BGLF4-regulated phosphoproteins involved in the nuclear pore complex. (XLSX)
