# Supplementary figures and images for: Phosphoproteomic Profiling Reveals Epstein-Barr Virus Protein Kinase Integration of DNA Damage Response and Mitotic Signaling
Source: PLoS Pathog. 2015 Dec 29;11(12):e1005346. doi: 10.1371/journal.ppat.1005346 (PMC4699913; doi:10.1371/journal.ppat.1005346)

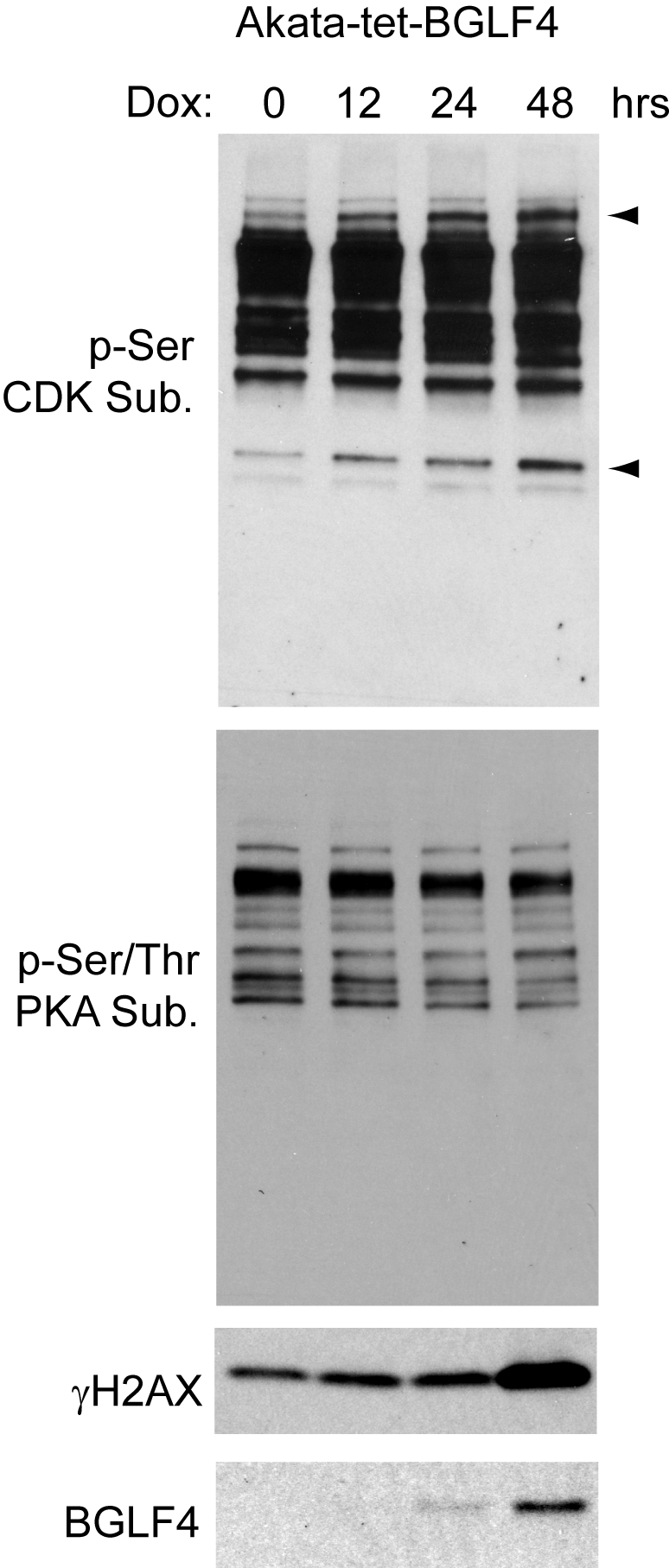

Supplement: S1 Fig — The motif specific antibodies recognize CDK or PKA substrates. (TIF) [file ppat.1005346.s001.tif]

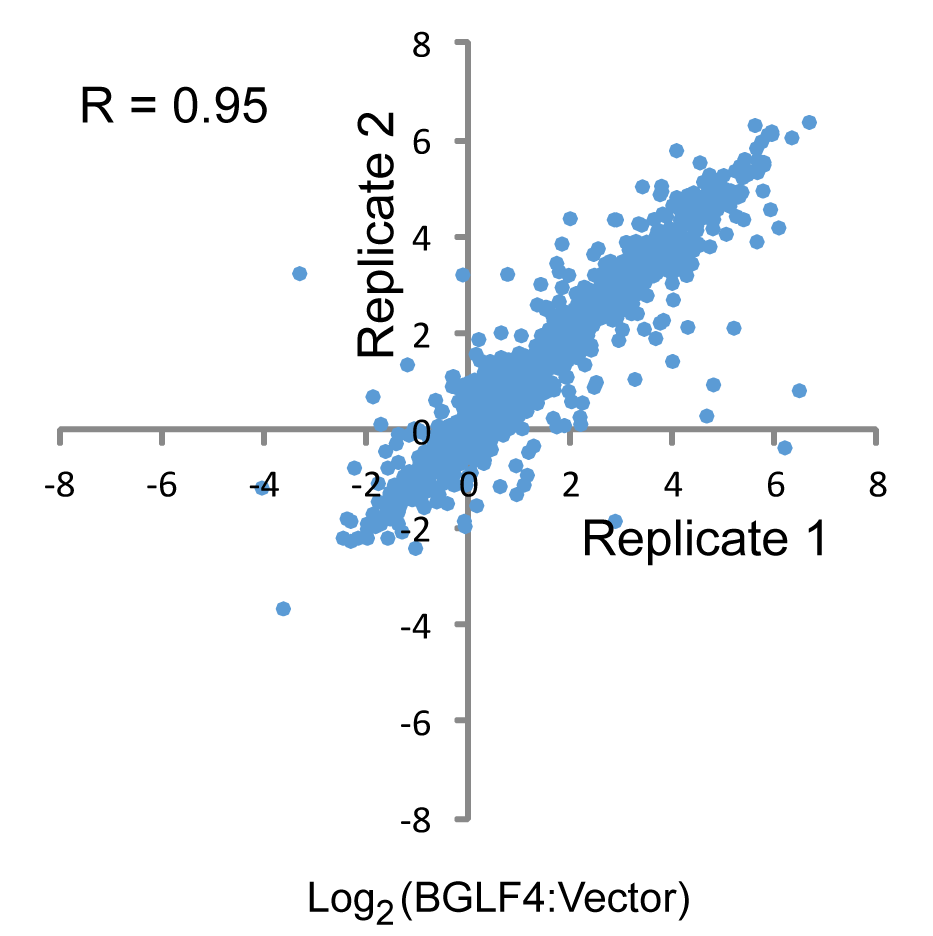

Supplement: S2 Fig — (TIF) [file ppat.1005346.s002.tif]

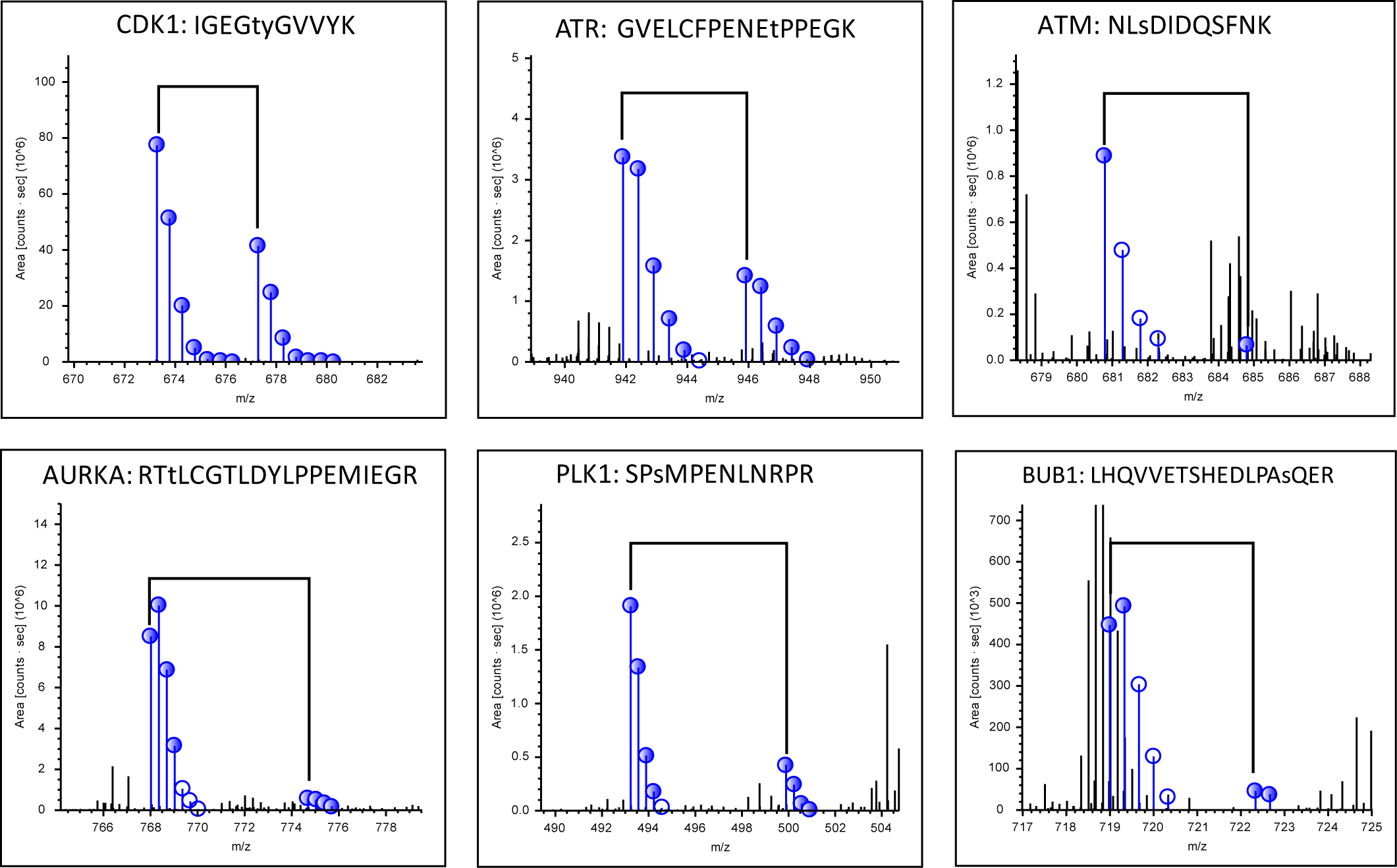

Supplement: S3 Fig — MS spectra showing the changes in the relative abundance of phosphopeptides [light (BGLF4) vs heavy (Vector)]. Phosphosites are labeled as lowercases. (TIF) [file ppat.1005346.s003.tif]

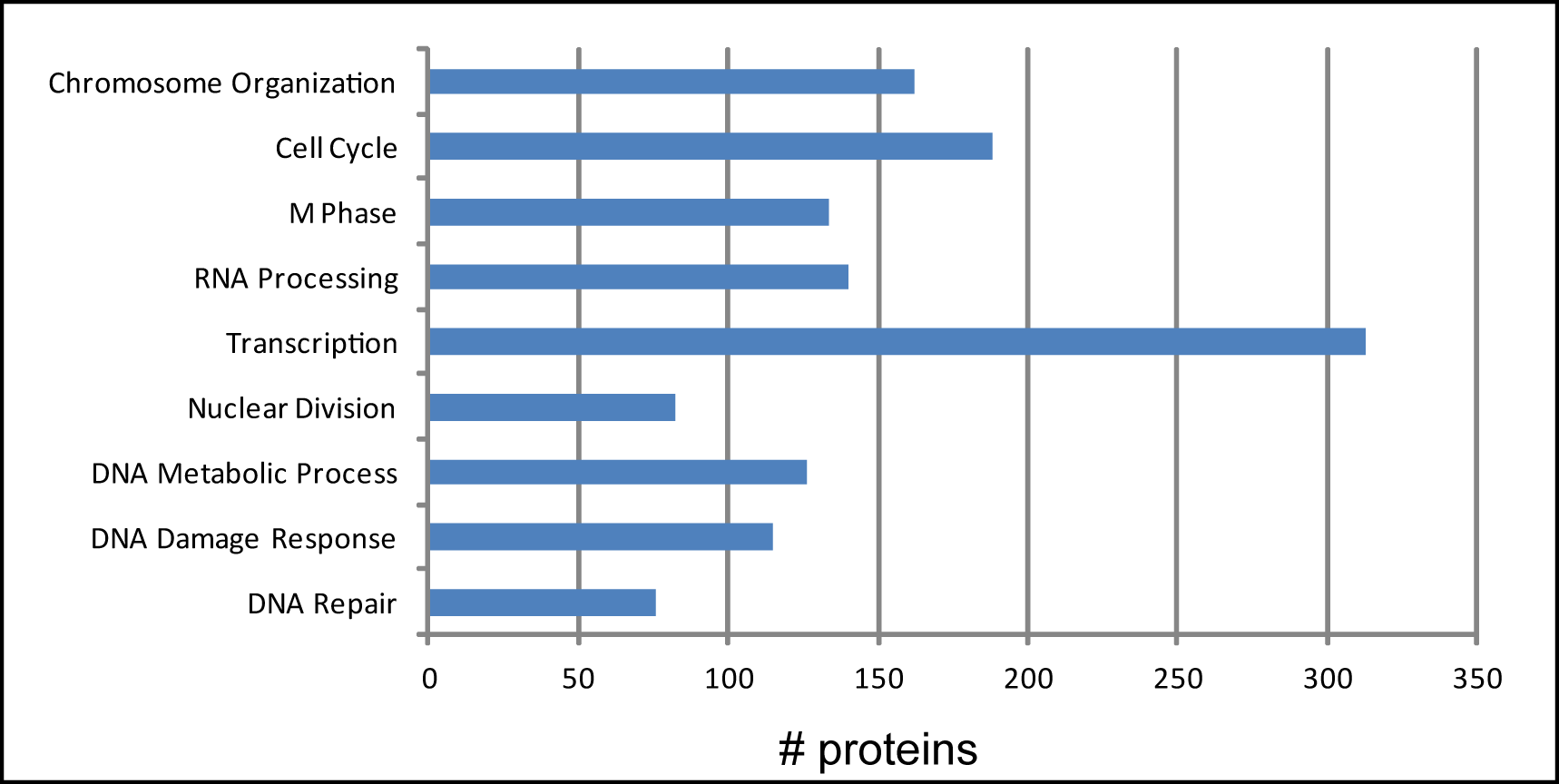

Supplement: S4 Fig — (TIF) [file ppat.1005346.s004.tif]

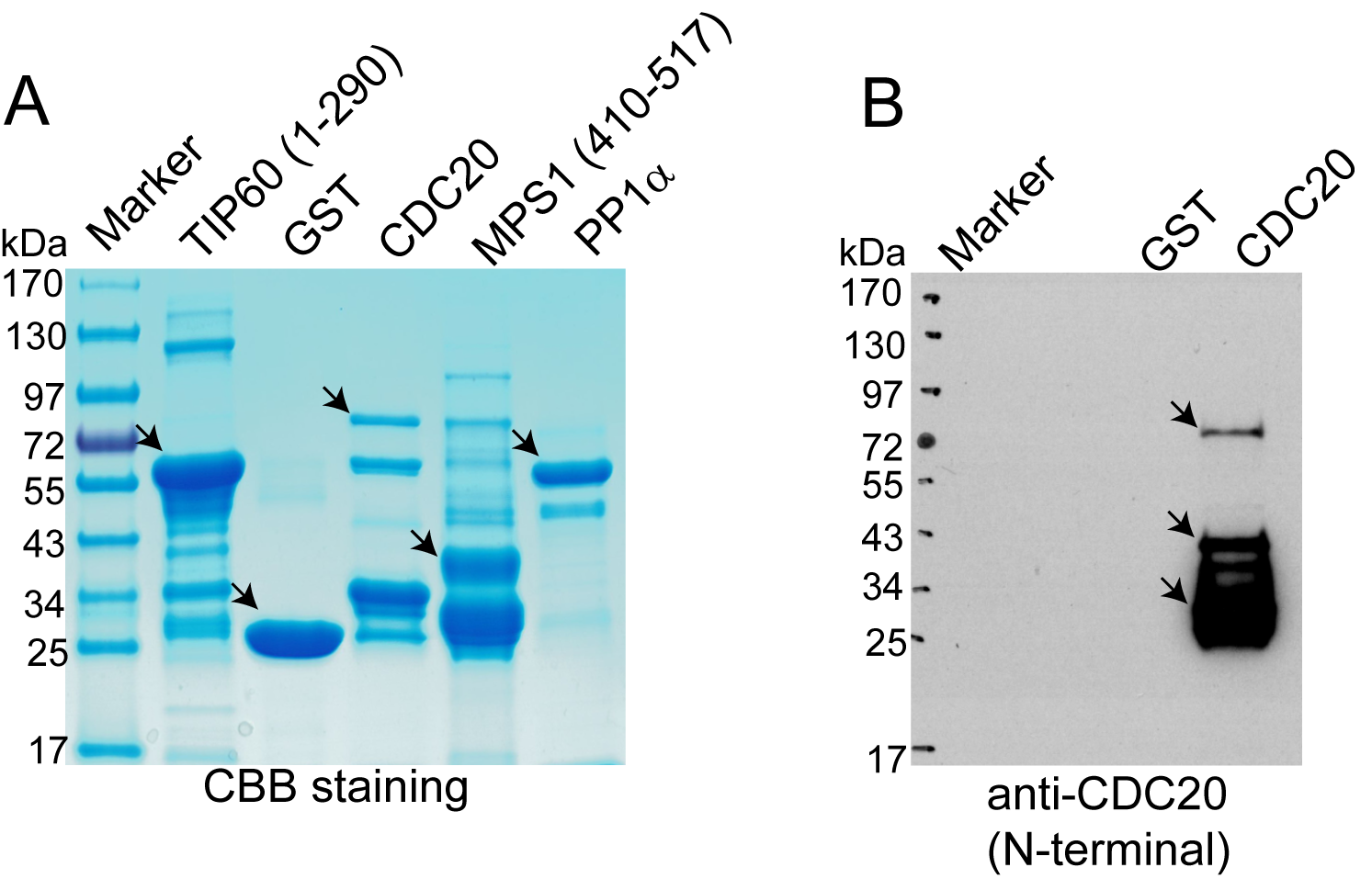

Supplement: S5 Fig — (A) Coomassie Brilliant Blue (CBB) staining showing the GST-tagged proteins and GST used in the assay. Arrows indicate the positions of GST and GST tagged proteins. (B) Immunoblot of purified GST-CDC20 reacted with anti-CDC20 (N-terminal) antibody. Arrows indicate the positions of GST-CDC20 and two major GST tagged N-terminal fragments of CDC20. (TIF) [file ppat.1005346.s005.tif]
